# IgD shapes the pre-immune naïve B cell compartment in humans

**DOI:** 10.3389/fimmu.2023.1096019

**Published:** 2023-01-26

**Authors:** Johannes Dirks, Oliver Andres, Luisa Paul, Georgi Manukjan, Harald Schulze, Henner Morbach

**Affiliations:** ^1^ Pediatric Immunology, Department of Pediatrics, University Hospital Würzburg, Würzburg, Germany; ^2^ Department of Pediatrics I, University Hospital Essen, University of Duisburg Essen, Essen, Germany; ^3^ Institute of Experimental Biomedicine I, University Hospital Würzburg, Würzburg, Germany

**Keywords:** B cell, IgD, IgM, immunoglobulin repertoire, B cell maturation, B cell receptor

## Abstract

B cell maturation and immunoglobulin (Ig) repertoire selection are governed by expression of a functional B cell receptor (BCR). Naïve B cells co-express their BCR as IgM and IgD isotype. However, the role of the additionally expressed IgD on naïve B cells is not known. Here we assessed the impact of IgD on naïve B cell maturation and Ig repertoire selection in 8 individuals from 3 different families with heterozygous loss-of-function or loss-of expression mutations in *IGHD*. Although naïve B cells from these individuals expressed IgM on their surface, the *IGHD* variant in heterozygous state entailed a chimeric situation by allelic exclusion with almost half of the naïve B cell population lacking surface IgD expression. Flow cytometric analyses revealed a distinct phenotype of IgD-negative naïve B cells with decreased expression of CD19, CD20 and CD21 as well as lower BAFF-R and integrin-β7 expression. IgD-negative B cells were less responsive *in vitro* after engaging the IgM-BCR, TLR7/9 or CD40 pathway. Additionally, a selective disadvantage of IgD-negative B cells within the T2 transitional and mature naïve B cell compartment as well as reduced frequencies of IgM^lo/-^ B cells within the mature naïve B cell compartment lacking IgD were evident. RNA-Ig-seq of bulk sorted B cell populations showed an altered selection of distinct V_H_ segments in the IgD-negative mature naïve B cell population. We conclude that IgD expression on human naïve B cells is redundant for generation of naïve B cells in general, but further shapes the naive B cell compartment starting from T2 transitional B cells. Our observations suggest an unexpected role of IgD expression to be critical for selection of distinct Ig V_H_ segments into the pre-immune Ig repertoire and for the survival of IgM^lo/-^ naïve B cells known to be enriched in poly-/autoreactive B cell clones.

## Introduction

Finely tuned signals transmitted by the B cell receptor (BCR) are essential for proper B cell development and the formation of a balanced immunoglobulin (Ig) repertoire (1). Surface expression of different BCR isotypes is differentially regulated with IgM starting early in the bone marrow and IgD peaking during more mature B cell stages. Naïve B cells express their BCR as IgM and IgD isotype, which share identical specificities within the same cell but differ in the Fc-region (2). IgM and IgD functions in B cells have long been considered as redundant, since either IgM- or IgD-deficient mice did not show major alterations of B cell development and/or function (3–5). Indeed, IgD could substitute for absent IgM function in developing B cells (5). Also, human individuals from one pedigree with a heterozygous *IGHD* variant that abrogated IgD expression in half of the naïve B cells did not reveal a major impact on B cell homeostasis or memory formation (6). However, lack of IgD expression in B cells from IgD knock-out mice impaired affinity maturation and in heterozygous mice IgD expressing cells outcompeted IgD-negative cells within the naïve B cell pool of (4, 7). Therefore, it was speculated whether IgD functions as an optimized BCR facilitating B cell recruitment into germinal center (GC) reactions by mechanisms that remain yet incompletely understood.

Former studies in BCR transgenic mouse models that established the concept of functional anergy as mechanism of peripheral B cell tolerance already indicated downregulation of IgM but not IgD in anergic B cells (8). Furthermore, autoreactive B cells in mice with an unmanipulated BCR repertoire displayed selective downregulation of IgM but not IgD (9–11). In line with this, B cells that express IgD, but little to absent IgM have been identified within the human mature naïve B cell compartment (“IgM^lo/-^ B cells” as well as “B_ND_ cells”), are functionally anergic and harbor high frequencies of autoreactive clones (12, 13). These and recent functional studies therefore suggest a differential impact of both, IgD and IgM, on maintaining peripheral B cell tolerance and fine-tuning the pre-immune BCR repertoire.

It is controversially debated by which molecular mechanisms the differential expression of IgM and IgD may impose anergy in mature B cells (2, 14, 15). Assessing BCR-signaling in GFP reporter mice with an unconstrained BCR repertoire background has revealed that follicular B cells downregulate IgM, but not IgD expression after sensing endogenous antigens (9, 16). In this model, IgD BCRs were less efficient than IgM at driving GFP reporter expression, suggesting that IgM, but not IgD BCRs are involved in sensing endogenous antigens and potentially anergy induction (9). Mechanistic insight into the distinct capabilities of IgM and IgD in sensing antigen came from studies using pro-B cells deficient for recombinase RAG2, adaptor SLP65 and the surrogate light chain component λ5 (“triple-deficient”) that were transduced with various BCR isotypes and specificities (17). In this model, IgM seems to be uniquely capable of responding to low-valency antigens whereas polyvalent antigens could activate both BCR isotypes. This difference was attributed to the more flexible IgD-specific hinge region. However, this observation has been challenged in a different experimental setting suggesting that both BCR isotypes might sense soluble monovalent antigen. IgD expression in this experimental setting has been shown to attenuate the response to endogenous antigen and promote their survival (18).

In addition to its function in regulation of anergy induction, signaling *via* IgM or IgD BCRs also seems to determine further differentiation fate decisions in B cells. In murine competition models, IgM-only B cells outcompeted IgD-only B cells in the B1a compartment, which is characterized by a highly self-reactive immunoglobulin repertoire (9). In contrast, IgD-only B cells preferentially populated the marginal zone B cell compartment, whose formation relies on weaker BCR signal strength (9). In addition, IgM favored differentiation of B cells into short-lived plasma cells, whereas activation through IgD excluded B cells from rapid extra-follicular plasma cell differentiation (9). It was assumed that these mechanisms may exclude anergic B cells that have downregulated IgM and are instructed to receive signals *via* IgD from uncontrolled differentiation into autoantibody-secreting plasma cells (9, 18). However, signaling through IgD may allow B cells to enter germinal center (GC) reactions (9, 18, 19). Even more, autoreactive B cells that have entered GC reactions after stimulation *via* IgD BCRs may lose autoreactivity by means of somatic hypermutation (SHM) and could be employed in further B cell responses towards foreign antigens (20, 21).

Altogether, observations from these experimental mouse models suggested that IgD mainly functions by controlling survival, differentiation and further use of low-affinity autoreactive B cells recognizing structurally distinct antigens (2, 15). Hence, expression of IgD might counter restrictive pressure on the naïve BCR repertoire that would otherwise impair the variability of the adaptive immune response.

Herein, we made use of the unique opportunity to assess the role of IgD in the naïve B cell compartment in humans by studying 8 individuals from three families, each carrying a distinct heterozygous *IGHD* variant. The chimeric setting in these individuals allowed the direct comparison of naïve B cells carrying either the wildtype (IgD-positive) or the mutant (IgD-negative) allele. An altered phenotype, selective disadvantage and a skewed immunoglobulin heavy chain repertoire was associated with loss of IgD expression on mature naïve B cells expressing the mutant *IGHD* allele, providing genetic evidence in humans that IgD is involved in shaping the pre-immune Ig-repertoire of naïve B cells.

## Material and methods

### Patients and controls

All patients and control individuals were followed at the University Children`s Hospital Würzburg. Control blood samples from adults have been donated on a voluntary basis. Control blood samples were also obtained as a part of routine blood draws in children who did not have infectious, hematological or immunological diseases. Signed informed consent was obtained by the legal representatives. The study was reviewed by the Research Ethic Committee of the University of Würzburg (Number 218/18) and conducted in accordance with the Declaration of Helsinki.

### Sample preparation

Blood samples were collected in EDTA tubes. Peripheral blood mononuclear cells (PBMCs) were isolated by Ficoll density-gradient centrifugation. Cells were stored in liquid nitrogen until use. B cells were purified using CD20-microbeads (Miltenyi Biotec). Flow cytometric cell sorting was performed using a FACSAria III (BD Biosciences).

### Genetic analysis

Genomic DNA was isolated from peripheral blood samples using QIAamp DNA Blood Kits (Qiagen). All exons of *IGHD* including exon/intron boundaries were amplified by PCR using the GoTaq DNA Polymerase (Promega). NG_001019.6 and IMGT gene tables were used as reference sequences. The following primers were used for PCR amplification of the *IGHD* gene: Exon1 Forward 5`-CTTGTCCTCAGAGCCTCCAG-3`, Exon1 Reverse 5`- TTCTGTCTTTGTGGTCAGGC-3`, Exon2 Forward 5`- CAGCCTCACCTGCACTTTTC-3`, Exon2 Reverse 5`-GGAGTTATGAAGGGCTGCCT-3`, Exon3 Forward 5`-ACTGCATGGTCATTAGCTGG-3`, Exon3 Reverse 5`-GGTGTTTTGATAGCCCAGGG-3`, Exon4 Forward 5`-TCTCGTTTGCTCTCCCCTG-3`, Exon4 Reverse 5`-CCCTTCTCCTTTCCTGTGG-3`, Exon5 Forward 5`-CCACAGGAAAGGAGAAGGGA-3`, Exon5 Reverse 5`-CACCCCTGCCTAGTATGGAT-3`, Exon6 Forward 5`- ATGAACAGAAAGACACGCCG -3`, Exon6 Reverse 5`- GCATTGACAAGAACCAGCCA -3`, Exon7 Forward 5`- TATGAGCAAGAGGGTGAGGCT-3`, Exon7 Reverse 5`- GGATCCCTGGACCAACTCTG-3`, Exon8 Forward 5`- TCATGACCAGGGAGCTTCTC-3`, Exon8 Reverse 5`- GTGGGTCCTTTCTGCTCTCTG-3`. Sanger sequencing of the PCR products was performed by a commercial provider (MWG eurofins) with the same primers as used for PCR amplification.

For analysis of *IGHD* allele distribution RNA obtained from CD19^+^CD27^-^IgM^+^ IgD^+^ or IgD^-^ B cells was reverse transcribed using iScript cDNA synthesis kit (Biorad, Hercules, CA, USA). The following primers were used in RT-PCR (GoTaq DNA Polymerase; Promega) for the amplification of the *IGHJ*/*IGHD Exon1* junction: IGHJ Forward 5`-CTGGTCACCGTCTCCTCAG-3` and IGHD Exon1 Reverse 5`- TTCTGTCTTTGTGGTCAGGC-3`. Sanger sequening of purified PCR products was performed by a commercial provider (MWG eurofins, Martinsried, Germany) with the following primer: IGHD 3`-Seq 5`-CCCATGTACCAGGTGACAGT-3`.

### Generation of IgD cDNA expression vectors and site-directed mutagenesis

For generation of a FLAG-tagged IgD molecule a nucleotide sequence encoding the FLAG-tag was cloned immediately 3´ of the *IGHD* constant region into the AbVec-huIgD vector containing a rearranged V(D)J-sequence (693-1-F06) (22). The c.16C>T (p.P6L) mutation was inserted into the vector using the Q5 Site-directed mutagenesis kit (NEB).

### Cell culture, transfection and immunoblot

IgD heavy chain and the corresponding kappa light chain plasmids were transiently co-transfected into HEK 293T cells using the polyethylenimine (PEI)-precipitation method. After two days cells were lysed in lysis buffer (50mM Tris, 1% NP-40, 2mM EDTA) including phosphatase inhibitor cocktail 2 (Sigma) and protease inhibitor (Roche). Total cell lysates were separated by SDS page, transferred to PVDF membranes. Membranes were blocked in 5% bovine serum albumin for one hour at room temperature and probed with specific primary antibodies (DYKDDDDK Tag (9A3) mouse mAb, Cell Signaling and β-actin mouse mAb 926-42212, LI-COR) overnight at 4°C, followed by staining with corresponding IRDye secondary antibodies (LI-COR) for one hour at room temperature. Specific band on immunblots were visualized using an Odyssey Infrared Imaging System (LI-COR).

### Flow cytometry

PBMCs were stained at 4°C for 30 min in 1X PBS 0.5% BSA with appropriate antibodies (**Supplementary Information**). Flow cytometry data was acquired on a FACSCanto II (BD Biosciences) for analyzed with FlowJo version 10 (Tree Star). Additionally, B cell subsets were sorted for immunoglobulin repertoire analysis on a FACSAria III (BD Biosciences).

### B cell stimulation

Total CD20^+^ B cells were plated at 100,000 cells/well in a 96-well-plate in RPMI 10% FBS and 2.5 μg/ml polyclonal F(ab’)_2_ anti-human IgM (Jackson Immunoresearch), 1.0 μg/ml CpG ODN2006 (*In vivo*gen) or 2 μg/ml Gardiquimod (*In vivo*gen). Expression of surface activation markers CD69 and CD86 was analyzed on gated CD19^+^CD27^-^ IgD^+^ or IgD^-^ cells after 48 hours by flow cytometry. For functional analysis of *IGHD* variants flow cytometrically sorted CD19^+^CD27IgM^+^IgD^+^ and CD19^+^CD27^-^IgM^+^IgD^-^ B cells were stimulated by anti-human IgM or anti-human IgD antibodies. Expression of CD69 was analyzed after 48 hours.

### Immunoglobulin gene sequencing and repertoire analysis

CD19^+^CD27^-^CD10^-^CD21^+^IgM^+^ IgD^+^ and IgD^-^ mature naïve B cells from four heterozygous *IGHD* carriers were sorted for immunoglobulin heavy chain (IgH) repertoire analysis. RNA from sorted cells was purified using RNeasy Micro Kit (Qiagen). IgH rearrangements were amplified using amplicon rescue multiplex PCR and sequenced by next generation sequencing (NGS) on an Illumina MiSeq platform (iRepertoire^®^, Huntsville, AL, USA). After initial filtering and mapping using iRepertoire^®^ algorithms, multiple sequence copies of unique sequences were counted as a single sequence. Resulting sequence data was analyzed using IMGT/HighV-QUEST, unproductive sequences were filtered out and only IGHM sequences were used for further analysis. Resulting files were analyzed for V-, D-, and J-segment usage, CDR3-length, and CDR3 biochemical characteristics using ARGalaxy (23). BCR clonotypes were defined by identical V-, D- and J- gene segment usage as well as identical CDR3 nucleotide sequence. For calculation of clonal diversity resultant Change-O databases were analyzed using Alakazam (24, 25). Standard settings were used for computation of the diversity scores (bootstrap n=200, ci=0.95). Loop properties were analyzed using AIMS (26). Ggplot2 was used for creating VJ-pairing dot plot. IgH sequences have been submitted to NCBI Sequence Read Archive (SRA) with the BioProject PRJNA895235.

### Statistical analysis

Statistical analyses were performed using GraphPad Prism software version 8.0. Data are expressed as scattered individual values and the mean ± SD. Either paired student’s 2-tailed t-test or One-way ANOVA with Turkey`s multiple comparison were used to compare data sets with either 2 or >2 continuous variables, respectively. One sample t-test was used the statistical significance between the ratio or the difference of two variables and 1 or 0, respectively. P-values less than 0.05 were considered statistically significant.

## Results

### Detection of heterozygous *IGHD* mutations in individuals displaying an altered B cell phenotype

**Figure 1:**
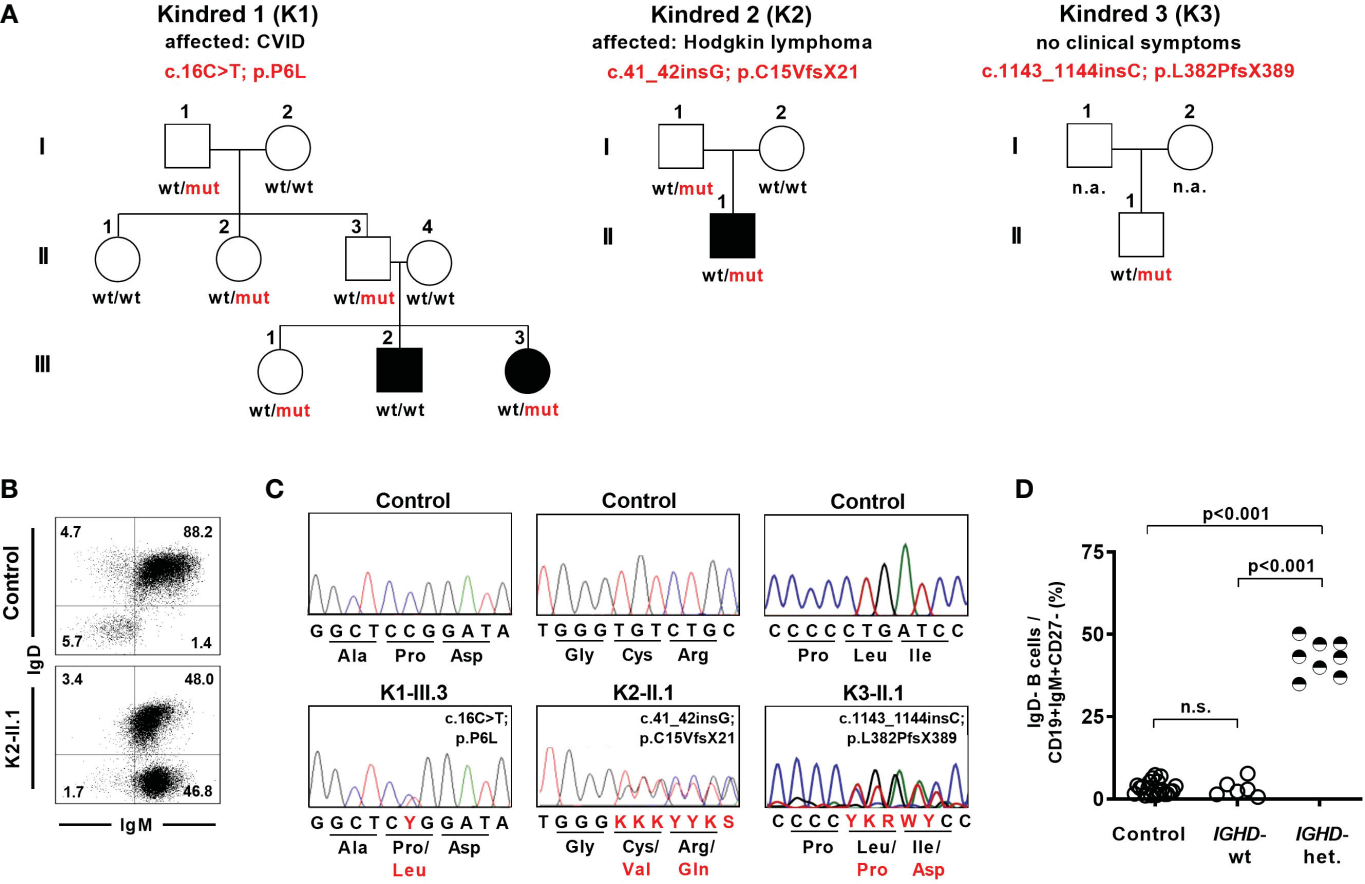
Lack of surface IgD expression in a fraction of naïve B cells in individuals carrying heterozygous *IGHD* variants. **(A)** Pedigree of individuals with heterozygous *IGHD* variants. Individuals affected by a disease are shown in black. Wild type (wt) and mutant (mut) describes the presence of respective *IGHD* alleles in each individual. **(B)** Representative dot plot showing IgD expression on CD19^+^CD27^-^IgM^+^ naïve B cells from a control individual as well as an individual carrying a heterozygous *IGHD* variant. **(C)** Representative sequencing chromatograms (*IGHD*, genomic DNA) of 3 individuals with different heterozygous *IGHD* variants and controls. **(D)** Compiled flow cytometric data showing the frequency of IgD-negative B cells within CD19^+^CD27^-^IgM^+^ naïve B cells in control individuals and family members without (*IGHD*-wt) or with (*IGHD*-het.) a heterozygous *IGHD* variant (One-way ANOVA with Turkey`s multiple comparison). n.s., not significant.

As part of a diagnostic work-up we identified eight individuals from three different families who showed an unusual pattern of IgD expression on naïve B cells and carried heterozygous *IGHD* variants (**Figure 1A**). The six-years-old index patient K1-III.3 was diagnosed with common variable immunodeficiency (CVID) and displayed loss of IgD expression in almost half of her IgM^+^CD27^-^ naïve B cells (**Figure 1B**). Genetic analysis revealed an *IGHD* missense variant (c.16C>T; p.P6L) in a heterozygous state in exon 1 (**Figure 1C**). This variant is very rare (allele count n=1 in the Genome Aggregation Database (gnomAD) v2.1.1; rs767340720; minor allele frequency 4.07 x 10^-6^) and concerns a position that is highly conserved in IgD between different species as wells as between different Ig isotypes in humans (**Supplementary Figure 1**). The variant has not been reported in a clinical context so far (ClinVar). Sanger sequencing of IgD mRNA expression in sorted B cell populations of the index patient revealed almost exclusively the wild type allele in IgD-positive and the variant allele in IgD-negative CD27^-^IgM^+^CD19^+^ naïve B cells (**Supplementary Figure 2**). We could not detect the variant allele in the index patient’s brother who also suffered from CVID. However, four additional family members carried the variant in a heterozygous state but were healthy and did not reveal any signs of antibody deficiency (**Figure 1A**). Whereas all of the individuals in this family who carried the heterozygous IGHD variant also showed loss of IgD expression on half of their naïve B cells, no altered B cell phenotype could be observed in those family members carrying the wild-type alleles (**Figure 1D**; **Supplementary Table 1**). Furthermore, most of the IgD-CD27- B cells in family members not carrying the *IGHD* variant and unrelated control individuals were isotype-switched and therefore resembled “double negative” B cells (**Supplementary Figure 3**). In contrast, the majority of IgD^-^CD27^-^ B cells in heterozygous carriers of the *IGHD* variant also expressed IgM and were therefore more likely to join the naïve B cell population.

Being aware of this peculiar IgD expression pattern in naïve B cells, we detected a heterozygous *IGHD* nonsense variant (c.41_42insG; p.C15VfsX21) in exon 1 in a 22-year-old patient suffering from Hodgkin’s lymphoma during immunologic follow-up three years after completion of chemotherapy (**Figures 1A–C**). This variant as well as the distinct IgD expression pattern on naïve B cells was also detected in his healthy father who did not show signs of antibody deficiency (**Supplementary Table 1**). An additional nonsense variant (c.1143_1144insC; p.L382PfsX389) in exon 7 was detected in a heterozygous state in a 7-year-old child who participated in a study for generation of B cell reference values and was excluded as an outlier due to an abnormal loss of IgD expression on half of the naïve B cell subsets (27) (**Figures 1A, C**). This child did neither suffer from immunodeficiency or autoimmunity nor did it display any signs of antibody deficiency (**Supplementary Table 1**). Both missense variants have not yet been reported.

Hence, the detected *IGHD* variants abrogated surface IgD expression in those B cells that have rearranged the variant allele, but did not segregate with disease. This chimeric situation with almost half of the naïve B cell population lacking surface IgD expression seemed to be a unique setting to assess the functional relevance of IgD on mature B cell differentiation as well as Ig repertoire selection in humans.

### Identified *IGHD* mutations are loss-of-expression or loss-of-functions variants

**Figure 2:**
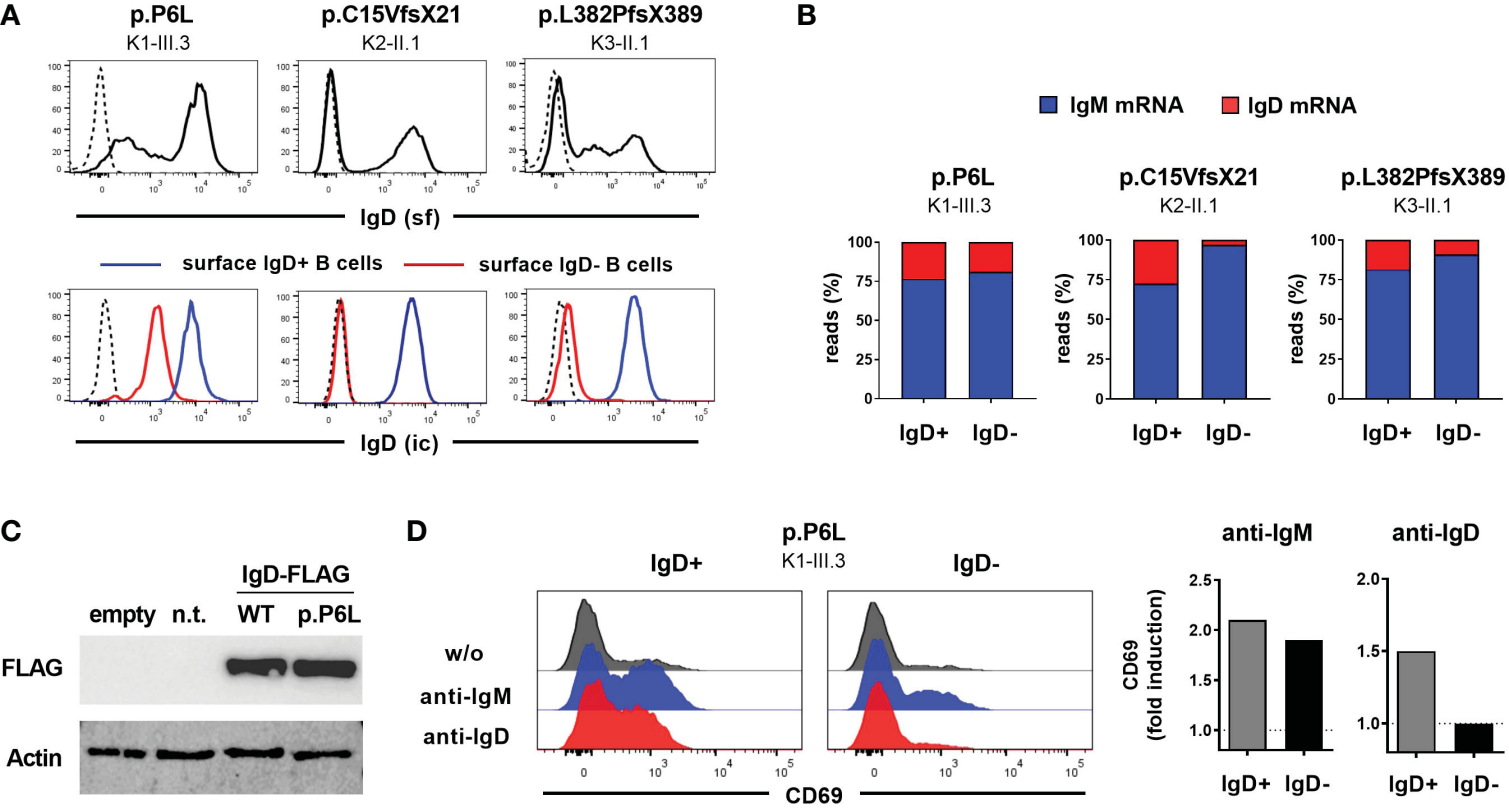
Characterization of *IGHD* variants reveal loss-of-expression or loss-of-function **(A)** Flow cytometric analysis of surface IgD expression on CD19^+^CD27^-^IgM^+^ naïve B cells from 3 individuals carrying different heterozygous *IGHD* variants (upper row). Intracellular IgD expression within surface IgD+ (blue line) or surface IgD- (red line) CD19^+^CD27^-^IgM^+^ naïve B cells from the same individuals (lower row). Dashed lines represent isotype controls. **(B)** Proportion of unique IgM or IgD sequence reads assessed by RNA-based IgH high-throughput sequencing within sorted CD19^+^CD27^-^IgM^+^CD10^-^ IgD^+^ or IgD^-^ mature naïve B cells from 3 individuals carrying different heterozygous *IGHD* variants **(C)** Immunoblot analysis of IgD Expression in 293T cells not transfected (n.t.) or transfected with an empty vector or an IgD-FLAG expression vector with wild type IgD sequence or the p.P6L variant. **(D)** Histograms showing flow cytometric analysis of CD69 expression on sorted IgD+ or IgD- CD19+CD27-IgM+ naïve B cells from an individual carrying the p.P6L *IGHD* variant stimulated *in vitro* with anti-IgM or anti-IgD for 48 hours. Fold induction (stimulated versus non-stimulated) of CD69 expression is shown on the right.

To further characterize the functional impact of the detected *IGHD* variants on IgD expression, we assessed surface and intracellular IgD expression in CD27^-^IgM^+^CD10^-^ mature naïve B cells from heterozygous carriers using flow cytometry. The heterozygous carriers of both nonsense variants displayed absence of surface IgD expression on the IgD-negative B cell population (**Figure 2A**). However, residual surface IgD expression on “IgD-negative” naïve B cells could be observed in the heterozygous carrier of the p.P6L variant (**Figure 2A**). This observation was even more pronounced when comparing intracellular IgD between surface IgD-positive and IgD-negative B cell populations (**Figure 2A**). Indeed, intracellular IgD expression could not be observed within surface IgD-negative B cells in carriers of both missense variants using a polyclonal anti-IgD antibody. Additionally, IgD RNA transcripts detected by IgH NGS were almost absent in the IgD-negative B cell subset of individual K2-II.1, suggesting that the p.C15VfsX21 allele was degraded by nonsense-mediated mRNA decay (**Figure 2B**). Hence, both p.C15Vfsx21 as well as p.L382PfsX389 can be regarded as loss-of-expression variants. In contrast, the “IgD-negative” B cell populations in the p.P6L carrier revealed normal IgD mRNA levels and reduced but residual intracellular IgD expression levels compared with the IgD-positive population (**Figures 2A, B**). To further corroborate this observation in a heterologous system, we generated FLAG-tagged IgD expression vectors containing the c.16C>T (p.P6L) IgD variant. Indeed, when transfecting 293T cells we could not detect significant differences in IgD expression levels between the wild-type and p.P6L variant by immunoblot staining, suggesting that this variant can become fully expressed (**Figure 2C**). In order to investigate the functional impact of the p.P6L variant, we assessed expression of activation markers on sorted surface IgD-positive and IgD-”negative” (with residual surface IgD expression) naïve B cells after *in vitro* stimulation with anti-IgM or anti-IgD (**Figure 2D**). Stimulation with anti-IgM did induce upregulation of CD69 in both B cell populations. In contrast, IgD-positive but not IgD-”negative” B cells showed upregulation of CD69 after anti-IgD stimulation. Hence, despite residual expression of the pP6L variant, these IgD molecules do not seem to be functional.

### Reduced frequencies of IgM^lo/-^ B cells within the mature naïve B cell compartment lacking IgD

**Figure 3:**
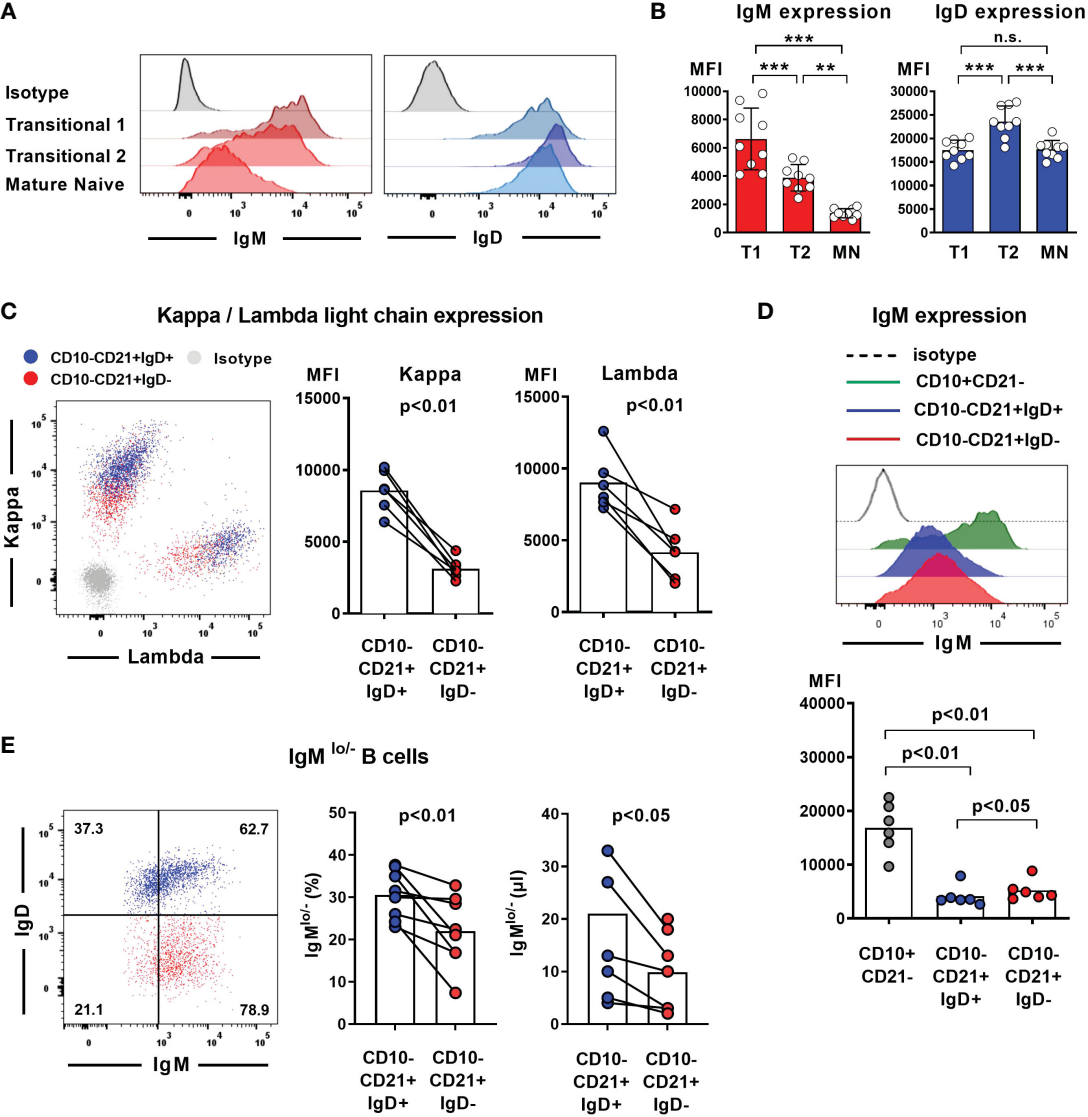
Loss of IgD expression is not compensated by upregulation of IgM expression in IgD-negative B cells **(A)** Representative histograms of IgM and IgD surface expression in different peripheral blood B cell subsets analyzed by flow cytometry (CD19^+^CD27^-^CD10^+^CD21^-^ transitional 1 (T1) B cells, CD19^+^CD27^-^CD10^+^CD21^+^ transitional 2 (T2) B cells, and CD19^+^CD27^-^CD10^-^CD21^+^ mature naive (MN) B cells, **(B)** Compiled flow cytometric data of IgM and IgD expression levels (mean fluorescence intensity, MFI) on different peripheral blood B cell subsets derived from 9 individuals. (**, p < 0.01; ***, p < 0.001; n.s., not significant; One-Way-ANOVA with Turkey`s multiple comparison test). **(C)** Representative dot blot showing surface expression of kappa and lambda light chain on IgD-positive (blue) or IgD-negative (red) CD19^+^CD27^-^CD10^-^CD21^+^ mature naïve B cells. Compiled data on expression levels are shown on the right (paired Student`s t-test). **(D)** Representative histograms obtained from flow cytometric analysis showing IgM expression levels on CD19^+^CD27^-^CD10^+^CD21^-^ transitional 1 B cells as well as IgD^+^ or IgD^-^ CD19^+^CD27^-^CD10^-^CD21^+^ mature naïve B cells. Compiled data from 6 individuals carrying different *IGHD* variants is shown in the lower panel (mean fluorescence intensity, MFI; One-way ANOVA with Turkey`s multiple comparison). **(E)** Representative dot blot showing the frequency of IgM^lo/-^ B cells within IgD^+^ (blue) or IgD^-^ (red) CD27^-^CD10^-^CD21^+^ mature naïve B cells from an individual carrying a heterozygous *IGHD*-variant. Compiled data from individuals with different *IGHD*-variants showing frequencies (left) and absolute counts (right) of IgM^lo/-^ B cells (paired Student`s t-test).

Absence of IgD on naïve B cells seems to be compensated by upregulation of IgM expression in some mouse strains that carry distinct *IGHD* variants. We therefore aimed at assessing IgM expression level within the heterozygous carriers of the *IGHD* variants. Since IgM and IgD expression levels are differentially regulated during B cell development, we first explored the level of these BCR isotypes on different naïve peripheral blood B cell subsets from control individuals (**Figures 3A, B**). IgM expression was highest on transitional 1 (T1) B cells and decreased during differentiation towards transitional 2 (T2) and mature naïve B cells. Conversely, transitional 2 (T2) showed highest expression levels of IgD. We then proceeded to analyze BCR expression levels on B cells from heterozygous *IGHD*-carriers. The mean level of kappa and lambda light chain on the IgD-negative B cell population was significantly reduced and reached half of that observed in the corresponding IgD-positive B cell population suggesting that loss of IgD expression is not grossly compensated by upregulation of IgM (**Figure 3C**). Indeed, both IgD-positive and IgD-negative mature naïve B cells showed downregulation of IgM expression compared to the matched transitional B cell subset from the same donor (**Figure 3D**). However, mean IgM expression levels were significantly higher on the IgD-negative mature naïve B cell population compared to the IgD-positive counterpart (**Figure 3D**). The maximum IgM expression levels of IgD-negative B cells did not exceed that of IgD-positive B cells and reduced frequencies of B cells with lower IgM levels rather seemed to account for the higher IgM expression levels in the IgD-negative subset (**Figure 3E**). Since mature naïve B cells display a broad range of IgM expression levels with IgM^lo/-^ B cells known to contain increased frequencies of autoreactive clones, we compared the frequency of IgM^lo/-^ B cells between the IgD-positive and IgD-negative population. Indeed, the frequency of IgM^lo/-^ B cells was significantly lower in the IgD-negative B cell population (**Figure 3E**). Taken together, these findings imply that increased IgM-expression levels in the absence of IgD might rather be accounted for by a selective loss of IgM^lo/-^ B cells and not by upregulated IgM expression that in general compensates for loss of IgD expression.

### Reduced responsiveness of naïve B cells lacking IgD towards BCR-, TLR7/9- or CD40-stimulation

**Figure 4:**
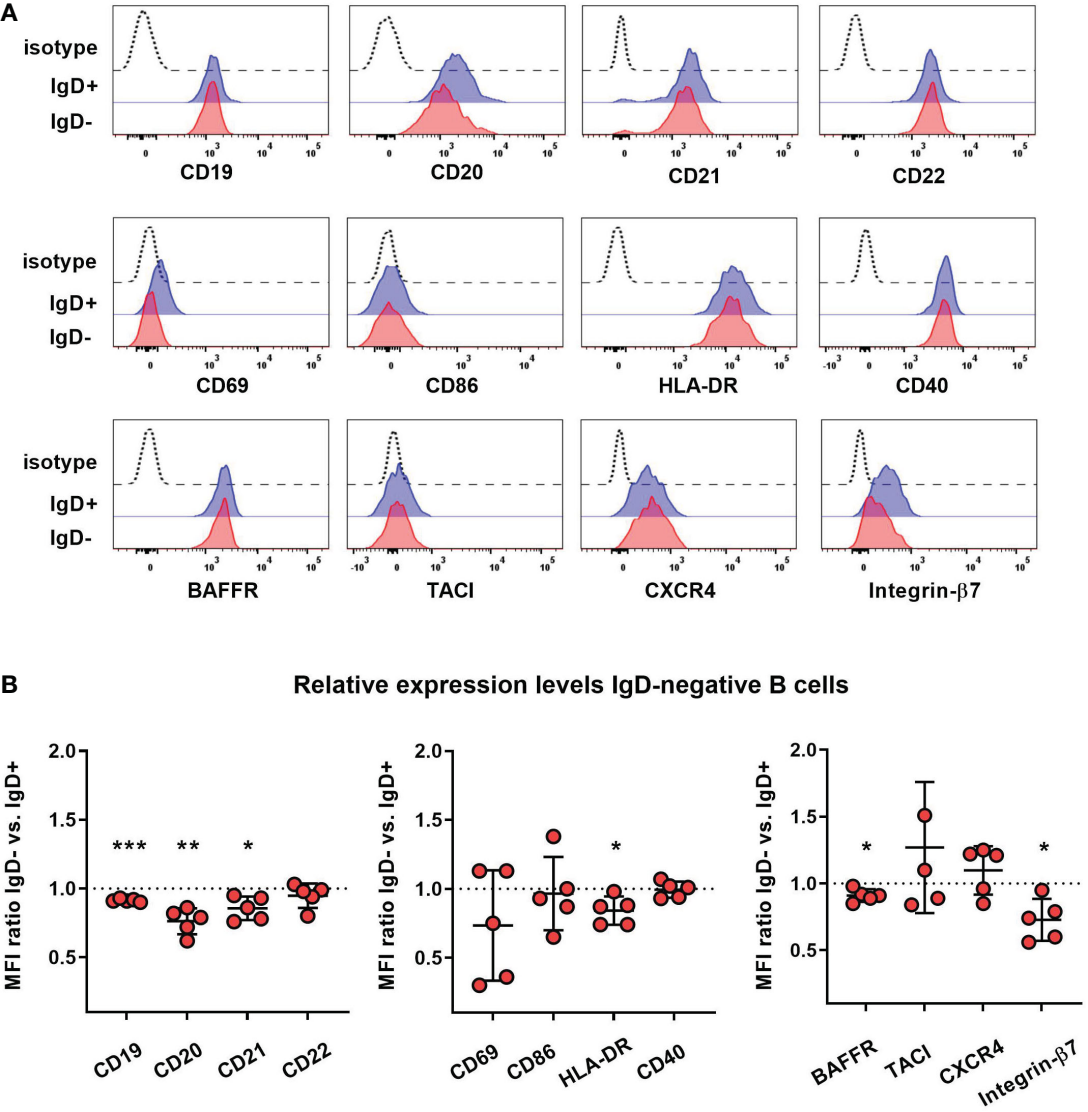
Reduced expression of co-stimulatory molecules on IgD-negative naïve B cells **(A)** Representative histograms obtained from flow cytometric analysis of IgD^+^ (blue line) or IgD^-^ (red line) CD19^+^CD27^-^IgM^+^ naïve B cells from an individual carrying a heterozygous *IGHD* variant showing expression of different markers. Dashed lines represent isotype control. **(B)** Relative expression levels of each marker within 5 different individuals carrying heterozygous *IGHD* variants is shown as IgD^+^/IgD^-^ ratio (mean fluorescence intensity, MFI; *, p < 0.05; **, p < 0.01; ***, p < 0.001 one sample t-test with hypothesized mean of 1).

**Figure 5:**
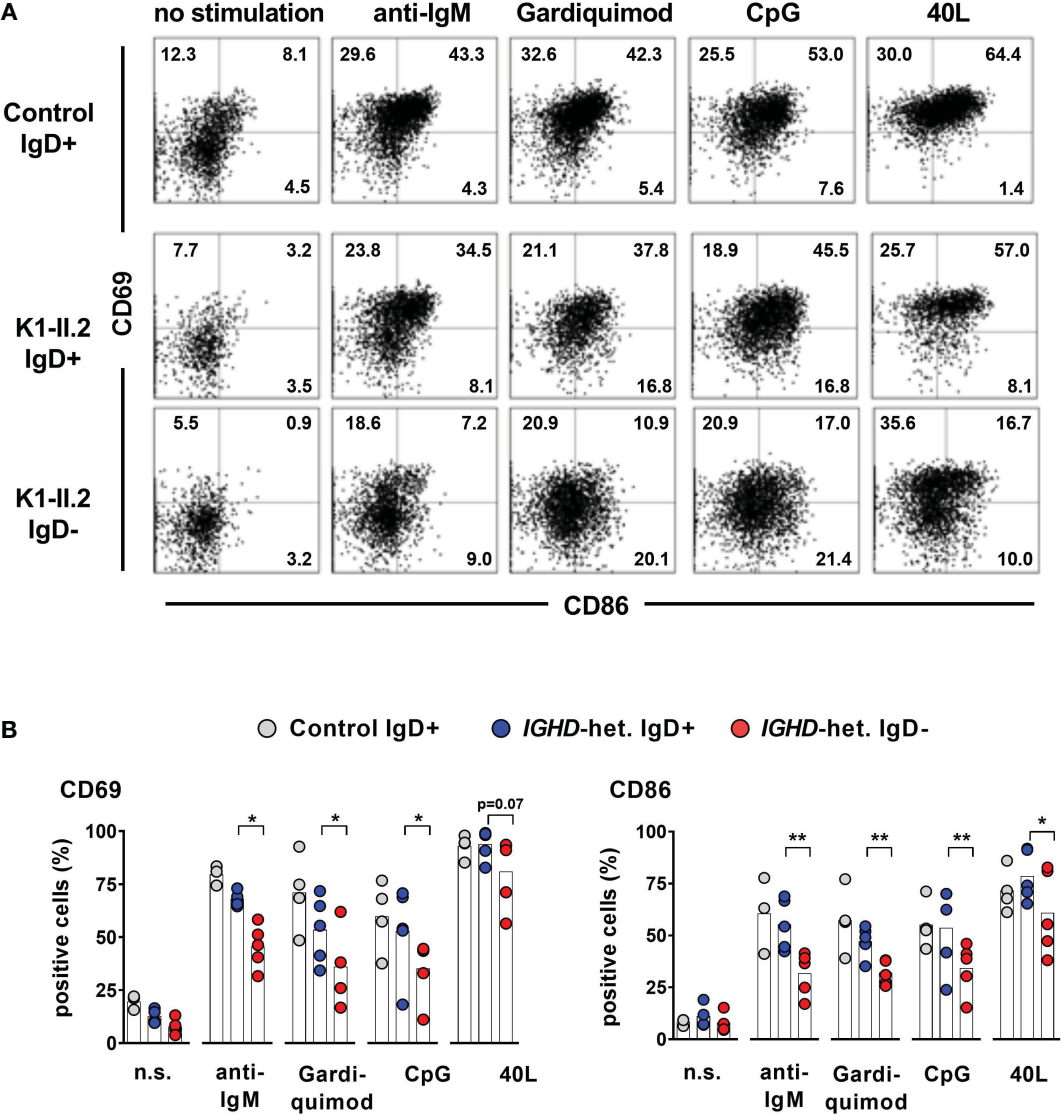
Impaired activation of IgD-negative naïve B cells after stimulation by IgM-BCR-, TLR7/9- or CD40 ligands **(A)** Representative dot blots showing expression of CD69 and CD86 on CD19^+^CD27^-^IgM^+^ IgD^+^ or IgD^-^ naive B cells stimulated or not with F(ab)_2_ anti-IgM, Gardiquimod (TLR7), CpG (TLR9) or CD40L for 48 hours from a control individual (upper row) or an individual carrying a heterozygous *IGHD* variant. **(B)** Compiled data from 4 control individuals as well as 5 individuals carrying different *IGHD* variants (paired Student`s t-test; **, p < 0.01; *, p < 0.05).

To assess whether lack of IgD expression might affect B cell maturation, we first assessed the phenotype of IgD-negative naïve B cells by flow cytometry and compared this to the matched IgD-positive B cell population within five individuals with different *IGHD* variants. The IgD-negative population revealed a reduced surface expression levels of CD19, CD20 as well as CD21, all of which are involved in amplifying BCR signals (**Figures 4A, B**). However, the expression levels of the rather inhibitory molecule CD22 were not different between both cell populations (**Figures 4A, B**). IgD-negative B cells did not show any signs of activation as evidenced by the lack of upregulation of CD69, CD86 or HLA-DR. Chemokine receptor expression patterns were altered in IgD-negative B cells with reduced levels of BAFFR and slightly increased CXCR4, but decreased expression of gut-homing integrin-β7. In contrast, expression levels of the co-stimulatory molecule CD40 as well as TACI did not differ between both subsets (**Figures 4A, B**). Overall, the phenotype of the IgD-negative naïve B cell population potentially indicated restricted survival signals and reduced ability to get activated. To functionally test this hypothesis, we next assessed IgD-positive and IgD-negative naïve B cell populations for their ability to respond to various activation signals *in vitro*. Upregulation of activation markers CD69 and CD86 on IgD-positive naïve B cells from heterozygous *IGHD* variant carriers after *in vitro* engagement of BCR (anti-IgM), TLR7 (Gardiquimod), TLR9 (CpG) or CD40 (CD40L) fell in the range of naïve B cells from unrelated healthy control individuals (**Figures 5A, B**). However, compared to IgD-positive B cells the matched IgD-negative naïve B cell population from heterozygous *IGHD* variant carriers showed a reduced upregulation of CD69 and CD86 after stimulation with any of these agonists (**Figures 5A, B**). Hence, naïve B cells that lack IgD expression *in vivo* do present a state of reduced responsiveness towards different stimuli *in vitro*.

### Selective disadvantage of IgD-negative B cells within the T2 transitional and mature naïve B cell compartment

**Figure 6:**
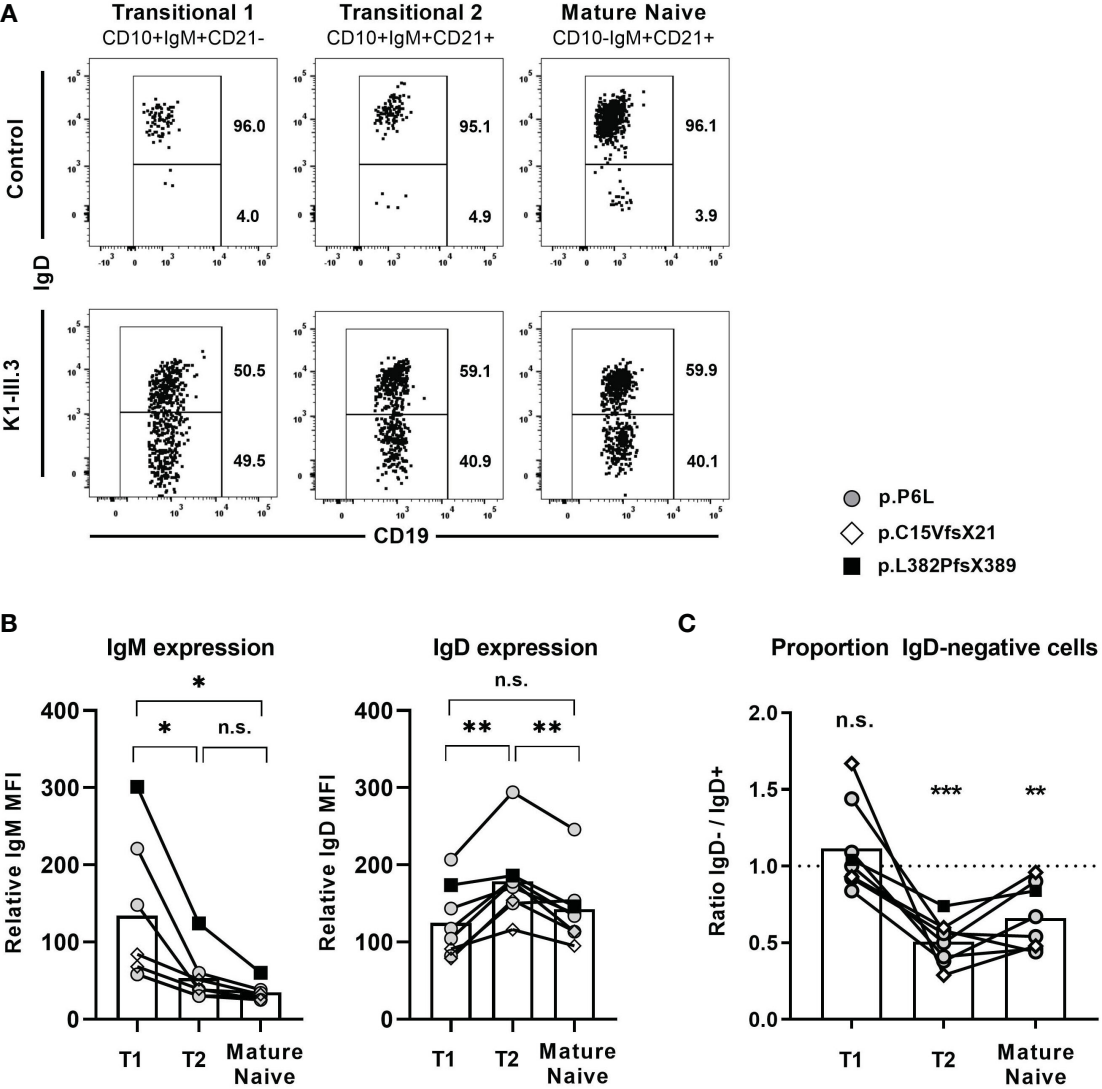
IgD-positive B cells outcompete IgD-negative B cells within the naïve B cell compartment **(A)** Representative dot blot obtained from flow cytometric analysis of CD19^+^ peripheral blood B cells from a control individual and an individual carrying a heterozygous *IGHD* variant showing the distribution of IgD^+^ and IgD^-^ cells within CD27^-^IgM^+^CD10^+^CD21^-^ transitional 1 B cells, CD27^-^IgM^+^CD10^+^CD21^+^ transitional 2 B cells and CD27^-^IgM^+^CD10^-^CD21^+^ mature naive B cells. **(B)** Compiled flow cytometric data of IgM and IgD surface expression levels in IgD^+^ transitional 1 (T1), transitional 2 (T2) and mature naïve IgD+ B cells in 8 individuals carrying different IGHD variants. The relative mean fluorescence intensity (MFI) was calculated as IgD or IgM MFI on different B cell subsets divided through the MFI on CD19^-^ non-B cells (background). **(C)** The distribution of IgD- B cells within each B cell subset was analyzed as ratio IgD-/IgD^+^ cells. (*, p < 0.05; **, p < 0.01; ***, p < 0.001; n.s., not significant; **(B)** One-Way-ANOVA with Turkey`s multiple comparison test; **(C)** one sample t-test with hypothesized mean of 1).

We next explored whether loss of IgD expression might also impair survival and homeostasis of naïve B cells. For this, we assessed the proportion of IgD-negative B cells within distinct naïve B cell subsets for all eight individuals carrying a heterozygous *IGHD* variant. IgD was expressed in almost all T1 and T2 as well as mature naïve B cell subsets of unrelated control individuals (**Figure 6A**). The expression levels of surface IgM and IgD within these IgD-positive B cell subsets of these individuals showed a similar pattern as observed in control individuals (**Figure 6B**; **Figures 3A, B**). The frequency of IgD-negative and IgD-positive B cells was similar within T1 B cells in the heterozygous carriers, suggesting no selective advantage to the expression of IgD at this B cell stage (**Figure 6C**). However, the ratio of IgD-negative to IgD-positive B cells within T2 B cells was shifted to the disadvantage of IgD-negative cells (**Figure 6C**). This shift was still present in the mature naïve B cell subset (**Figure 6C**). The pattern was evident in all analyzed individuals and not associated with a distinct *IGHD* variant, however, differences in the extent of skewing were noted between individuals (**Figure 6C**). These observations suggest a selective disadvantage of B cells that do not express IgD, which comes into play at the transition of T1 to T2 B cells when IgD is upregulated for the first time.

### Altered selection of distinct V_H_ segments in IgD-negative B cells

We next asked whether an undirected and BCR-independent cell loss or a selective pressure that impinges on certain BCRs might cause the disadvantage of naïve B cells that do not express IgD. We therefore assessed the IgH repertoire using next-generation sequencing within bulk sorted IgD-positive and IgD-negative CD19^+^CD27^-^IgM^+^CD10^-^ mature naïve B cell populations from four individuals carrying different heterozygous *IGHD* variants (**Supplementary Table 2**).

**Figure 7:**
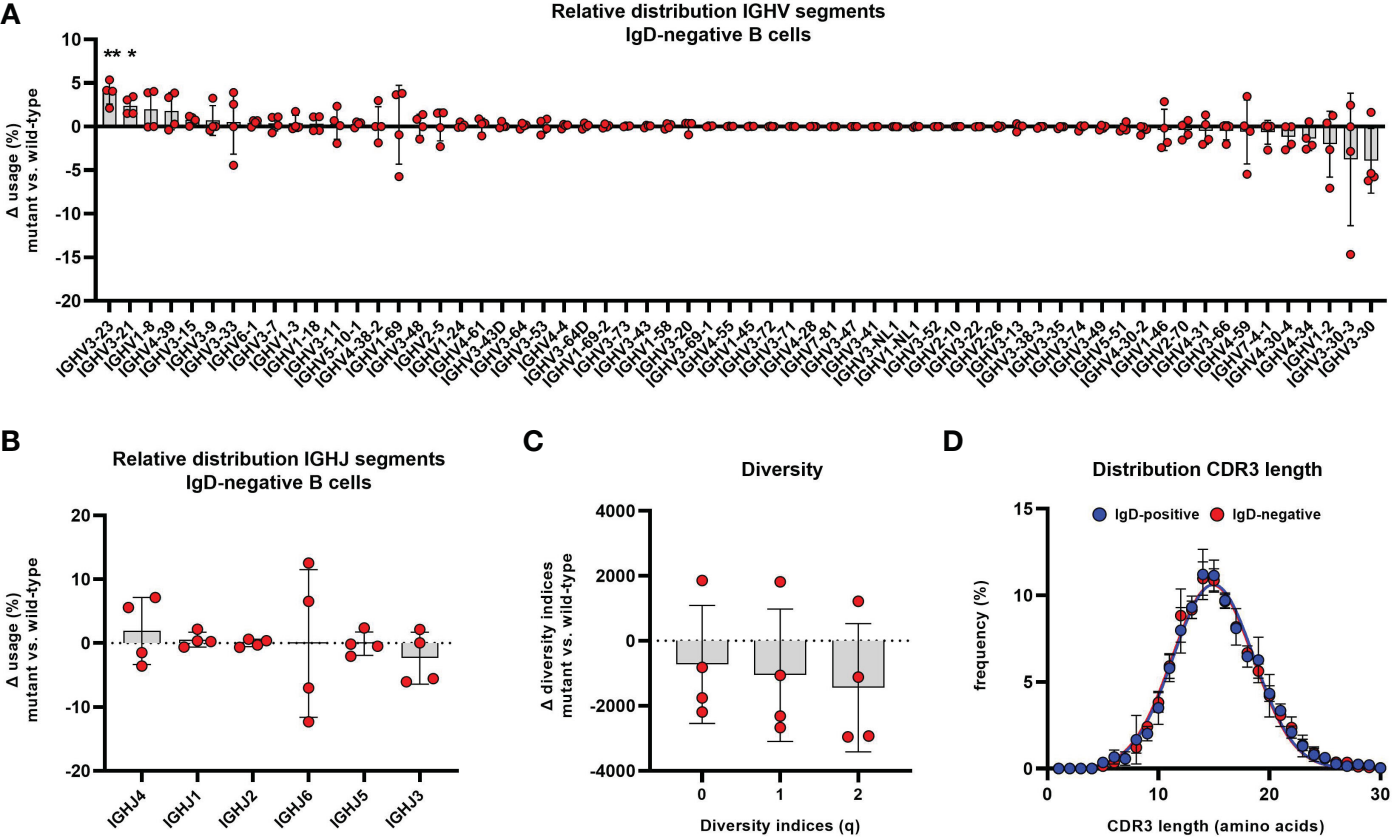
Altered distribution of V_H_ segments in the IgD-negative mature naïve B cell population Distribution of different V_H_ **(A)** and J_H_ **(B)** segments used in CD19^+^CD27^-^CD21^+^CD10^-^IgM^+^IgD^-^ mature naïve B cells is shown as relative difference compared to the matched IgD^+^ counterpart. Symbols represent the Δ frequency IgD^-^/mutant versus IgD^+^/wild-type from individual samples and bars the mean ± SEM. V_H_ and J_H_ segments are ordered on the x-axis according to their mean relative difference (*, p < 0.05; **, p < 0.01; one sample t-test with hypothesized mean of 0). **(C)** Representation of species richness (q=0), Shannon diversity index (q=1) and Simpson diversity index (q=2) is shown as difference between IgD^-^ and IgD^+^ B cell subsets. Symbols represent the Δ value IgD^-^/mutant versus IgD^+^/wild-type of each parameter from individuals samples and bars the mean ± SEM. **(D)** Distribution of CDR3 IgH length in IgD^+^ (blue) and IgD^-^ (red) mature naïve B cells. Each symbol represents the mean frequency ± SEM of a distinct CDR3 length.

Differences could be observed within the distribution of V_H_ segments, which indicated subtle, but significant alterations of IgH repertoire selection within IgD-negative B cells. The frequency of B cell clones using V_H_ segments 3-23 and 3-21 were significantly overrepresented in the IgD-negative compared to the IgD-positive compartment (**Figure 7A**). This observation was obvious in each analyzed individual and could not be accounted for by an extreme IgH repertoire skewing within one or few distinct individuals. We did not observe alterations in the distribution of J_H_ segments between both populations (**Figure 7B**). Also, B cell clones using V_H_3-23 or V_H_3-21, which were overrepresented in the IgD-negative B cell population, used different J_H_ segments. In detail, rearrangements between V_H_3-21 and J_H_3 or J_H_4 and between V_H_3-23 and J_H_4 or J_H_5 in particular accounted for this alterations (**Supplementary Figure 4**). In addition, V_H_3-7 and J_H_4 or J_H_5 rearrangements were also significantly overrepresented in the IgD-negative B cell population (**Supplementary Figure 4**). In contrast, B-cell clones using V_H_3-30 with either J_H_3 or J_H_4 as well as V_H_4-34 with either J_H_4 or J_H_5 rearrangements appeared to be underrepresented in the IgD-negative population (**Supplementary Figure 4**). This trend did not reach statistical significance due to individual outliers. However, when assessing the difference of V_H_ usage between both populations, it was interesting to observe, that V_H_3-34, V_H_1-2 as well as V_H_3-30-3 and V_H_3-30 were the V_H_ segments that were ranked in being most underrepresented in the IgD-negative B cell populations (**Figure 7A**). Of note, V_H_3-30 and V_H_3-30-3 as well as V_H_4-34 have been reported to be highly enriched in B cells that express IgD, but low to absent IgM in humans (12, 28–30). To explore whether the altered usage of VH segments may be associated with restricted diversity of the IgD-negative B cell population, we assessed the clonal diversity of both B cell subsets using the method of Hill over a range of Hill numbers. Although the IgD-negative B cell subset in three out of the four individuals showed lower species richness (q=0), Shannon entropy (q=1) as well as inverse Simpson index (q=2) compared to the matched IgD-positive population, this pattern was reversed in the fourth analyzed individual (**Figure 7C**).

We then assessed the IgH repertoire of both B cell populations for distinct differences in the CDR regions that are particularly involved in antigen binding and further repertoire selection. The overall CDR3 length was not different between both populations (**Figure 7D**). Also, DH segment reading frames that may affect binding characteristics of the CDR3 region did not differ between the IgD-positive and IgD-negative B cell population (**Supplementary Figure 5**). Additionally, the biochemical properties of CDR regions as defined by loop charge and hydrophobicity indicated slight differences between the IgD-negative and IgD-positive B cell population within some individuals but did not demonstrate any uniformly altered direction within the analyzed cohort (**Supplementary Figure 6**). Furthermore, the sequence diversity as quantified using Shannon entropy of both repertoires were similar between IgD-negative and IgD-positive B cell subsets (**Supplementary Figure 7**). Additionally, we could not identify a different pattern of interdependence of amino acids at residue positions belonging to CDR regions between both subsets3as assessed by the difference in mutual information (**Supplementary Figure 7**).

Taken together, these experiments reveal evidence that the IgH repertoire of IgD-negative mature naïve B cells is characterized by positive and negative selection of distinct V_H_ segments. As the detailed analysis of the biochemical properties of the CDRs did not show obvious differences this suggests that antigen binding by the highly variable regions may not be associated with altered selection of distinct V_H_ segments in IgD-negative B cells.

## Discussion

The main finding on the naïve B cell compartment in humans obtained from this cohort of individuals with heterozygous *IGHD* mutations revealed that IgD expression is redundant for generation of naïve B cell in general, but further shapes the naive B cell compartment starting from T2 transitional B cells on. These observations suggest IgD to be critical for selection of distinct Ig V_H_ segments into the pre-immune immunoglobulin repertoire and survival of IgM^lo/-^ naïve B cells known to be enriched in poly-/autoreactive B cell clones.

In our cohort we were able to describe different types of *IGHD* mutations that result either in IgD loss-of-expression or in residual expression associated with a loss-of-function. We could not find any association between the presence of any of the heterozygous *IGHD* mutations and a distinct clinical phenotype. Hence, the chimeric constellation of no more than half of the naïve B cells expressing surface IgD does not result in antibody deficiency due to haploinsufficiency. The same could be demonstrated in a previous study of individuals with a heterozygous nonsense mutation in *IGHD*, which also abrogated surface IgD expression in half of the naïve B cells (6). In detail, this study could not reveal evidence of impaired memory B cell or plasma cell formation from IgD-deficient B cells. However, immunoglobulin repertoire selection and clonal differentiation had not been assessed within this study. This could explain the differences to observations from mouse models, which suggest a role for IgD signaling in shunting autoreactive naïve B cells away from differentiation into short-lived plasma cells and supporting differentiation within germinal center trajectories (9, 18, 19, 31, 32). Lack of polymorphic differences between wildtype and mutant alleles in those individuals analyzed in our study made tracking of naïve B cells into the isotype-switched memory compartment difficult. Therefore, we particularly focused on the *in vivo* role of IgD on naïve B cell differentiation and generation of the pre-immune B cell repertoire.

The lack of IgD expression on naïve B cells in our study paralleled alterations of the B cell phenotype with lower expression of the co-receptors CD19, CD20 and CD21, but unaltered CD22 expression. On resting B cells, IgD resides in different protein islands than IgM and is in close proximity with the co-receptors CD19 and CD20, the latter organizing the IgD-nanocluster (33). Hence, lower expression of co-receptors on IgD-negative B cells could be explained by generally reduced IgD-BCR-complexes. Although IgM expression levels were significantly higher on IgD-negative compared to IgD-positive B cells (but did not reach the level of transitional B cells), upregulation of IgM did not completely compensate the loss of surface BCR expression levels by absence of IgD. This observation is in contrast to IgD- or Zfp318- knock-out mice, in which IgM is highly upregulated in the absence of IgD (4, 7, 34, 35). In contrast, surface IgD-deficient mice harboring an IgD Ile81Lys substitution that prevents folding of the IgD CH1 domain into the conformation needed to pair with immunoglobulin light chain showed unaltered surface IgM expression levels (18). Likewise, the IgD mutations described in our study allowed testing the impact of loss of IgD expression on naïve B cells independently of the inhibitory effects of IgD on surface IgM levels.

IgD-expressing B cells outcompeted IgD-deficient B cells in all individuals assessed in our study: This finding was most obvious at the stage of transitional T2 B cells, but was also evident within the mature naïve B cell compartment. The findings recapitulated recent observations in heterozygous IgD knock-out mice or in mixed bone marrow chimeras, in which loss of surface IgD expression conferred a selective disadvantage onto naïve B cells (6, 7, 18, 35). Generally decreased survival signals due to reduced expression levels of BCRs together with co-receptors and lower BAFF-R expression could account for this phenomenon in those individuals assessed in our study. However, upregulation of IgM compensated for loss of IgD expression in the murine IgD knock-out models. This suggests that rather functional differences in signaling outcome of both BCR isotype than overall BCR levels might have affected naïve B cell differentiation (9, 18). Indeed, observations from our study as well as other studies rather favor a model in which IgM and IgD BCRs respond differentially to certain antigens and by this shape B cell fate on a clonal level: 1. IgM is downregulated on naïve B cells after encountering endogenous antigens and IgM^lo/-^ B cells are enriched in autoreactivity (10, 11, 13, 16, 28, 36, 37). 2. IgD is less responsive than IgM after encountering endogenous antigens (9). 3. The range of IgM downregulation on maturing naïve B cells is restricted in the absence of IgD, resulting in the preferential loss of IgM^lo/-^ naïve B cells in our study. 4. The IgD-deficient naïve B cell compartment showed altered selection of distinct Ig V_H_ segments, some of which are associated with autoreactivity (e.g. VH4-34). Hence, these data may support the hypothesis that IgD expression on naïve B cells maintains the survival of autoreactive B cell clones that have not been removed by prior B cell tolerance checkpoints.

Differential responses to antigens elicited by IgM or IgD BCRs also seem to impact on further B cell fate decisions since signaling through IgD, but not IgM supports marginal zone B (MZB) cell differentiation, whereas the opposite favors differentiation into B1a cells (9, 17). In humans, B cell maturation seems to bifurcate from transitional T2 B cells that are selectively recruited into gut-associated lymphatic tissue and after selection/activation differentiate into (pre)MZB cells (38, 39). Our observation of a selective disadvantage of IgD-deficient B cells that particularly act on transitional T2 B cells as well as reduced expression of the gut-homing receptor integrin β7 on the IgD-deficient B cells may fit well in this model.

The B_ND_ cell pool in humans that express IgD but not IgM and primarily reside within mucosal tissues of the respiratory tract is highly enriched in autoreactive B cell clones (12, 28, 36). Additionally, a majority of B_ND_ cells display a stereotype Ig repertoire pattern with expression of VH3-30, VH3-30-3 or VH4-34 segments that do not show clonal overlap (28, 30). This led to the assumption that IgD-only cells may have been developed in a superantigen-driven reaction (29). In line with this, the binding specificity of autoantibodies directed towards distinct carbohydrates on erythrocytes (I/i antigen) is mediated by an intrinsically autoreactive framework region (FWR) 1 of the VH4-34 gene segment (40). Additionally, contributions from the FWR3 of VH3-30 have been accounted for its preferential selection into the IgD-only B cell pool (29). Interestingly, within our study, VH3-30, VH3-30-3 as well as VH4-34 were amongst those V_H_-segments that tended to be most counterselected in IgD-defcient B cells, supporting the hypothesis that IgD may be involved in controlling the selection of naïve B cells expressing autoreactive/stereotype BCRs.

In contrast to our observations, a recent study exploring B cell differentiation in four individuals with a heterozygous missense variant in *IGHD* that also abrogated surface IgD expression could not detect alterations in naïve B cell homeostasis (6). Indeed, B cells expressing the mutant or wild-type IgD allele were equally distributed within the mature naïve B cell compartment. A different functional effect of this mutation is unlikely to be the cause of these differences, as this mutation like the missense mutations assessed in our study resulted in the lack of IgD expression. Following the hypothesis that IgD is mainly involved in controlling distinct autoreactive B cell clones within the naïve B cell pool, individual differences within the composition of the pre-immune immunoglobulin repertoire may determine the extent of further negative selection that is imposed on IgD-deficient B cells. Indeed, although the Ig repertoire of IgM^lo/-^ B cells in humans did indicate lesser restriction in general, the burden of autoreactivity encoded within this repertoire (e.g. VH4-34) showed high interindividual differences (41).

By investigating human individuals carrying heterozygous mutations in *IGHD* that abrogate surface IgD expression we could demonstrate that IgD does not affect B cell development in general. However, loss of IgD expression on naïve B cells resulted in altered Ig repertoire selection that might affect survival of IgM^lo/-^ B cells potentially expressing autoreactive BCRs. Thus, finely tuned signals transmitted by IgM and IgD BCRs are essential for proper formation of a protective pre-immune immunoglobulin (Ig) repertoire that is tightly balanced between the strive for maximal diversity and the avoidance of harmful autoreactive BCRs.

## Data availability statement

The data presented in the study are deposited in the NCBI Sequence Read Archive repository, accession number: BioProject PRJNA895235.

## Ethics statement

The studies involving human participants were reviewed and approved by Ethikkommision Universität Würzburg. Written informed consent to participate in this study was provided by the participants’ legal guardian/next of kin.

## Author contributions

JD and HM designed the study and wrote the first draft of the manuscript. JD, OA, LP, GH, GM, HS and HM performed experiments and analyzed data. HM supervised the study. All authors contributed to the revision of the manuscript and approved the final version of the manuscript.

## References

[1] Meffre E O’Connor KC. Impaired b-cell tolerance checkpoints promote the development of autoimmune diseases and pathogenic autoantibodies. Immunol Rev (2019) 292:90–101. doi: 10.1111/imr.12821 31721234 PMC9145185

[2] Hobeika E Maity PC Jumaa H. Control of b cell responsiveness by isotype and structural elements of the antigen receptor. Trends Immunol (2016) 37:310–20. doi: 10.1016/j.it.2016.03.004 27052149

[3] Brink R Goodnow CC Crosbie J Adams E Eris J Mason DY. And d antigen receptors are both capable of mediating b lymphocyte activation, deletion, or anergy after interaction with specific antigen. J Exp Med (1992) 176:991–1005. doi: 10.1084/jem.176.4.991 1402669 PMC2119398

[4] Nitschke L Kosco MH Kohler G Lamers MC. Immunoglobulin d-deficient mice can mount normal immune responses to thymus-independent and -dependent antigens. Proc Natl Acad Sci U.S.A. (1993) 90:1887–91. doi: 10.1073/pnas.90.5.1887 PMC45985 8446604

[5] Lutz C Ledermann B Kosco-Vilbois MH Ochsenbein AF Zinkernagel RM Kohler G. IgD can largely substitute for loss of IgM function in b cells. Nature (1998) 393:797–801. doi: 10.1038/31716 9655395

[6] Nechvatalova J Bartol SJW Chovancova Z Boon L Vlkova M van Zelm MC. Absence of surface IgD does not impair naive b cell homeostasis or memory b cell formation in IGHD haploinsufficient humans. J Immunol (2018) 201:1928–35. doi: 10.4049/jimmunol.1800767 30143588

[7] Roes J Rajewsky K Immunoglobulin D. (IgD)-deficient mice reveal an auxiliary receptor function for IgD in antigen-mediated recruitment of b cells. J Exp Med (1993) 177:45–55. doi: 10.1084/jem.177.1.45 8418208 PMC2190865

[8] Goodnow CC Crosbie J Adelstein S Lavoie TB Smith-Gill SJ Brink RA. Altered immunoglobulin expression and functional silencing of self-reactive b lymphocytes in transgenic mice. Nature (1988) 334:676–82. doi: 10.1038/334676a0 3261841

[9] Noviski M Mueller JL Satterthwaite A Garrett-Sinha LA Brombacher F Zikherman J. IgM and IgD b cell receptors differentially respond to endogenous antigens and control b cell fate. Elife (2018) 7. doi: 10.7554/eLife.35074 PMC5897097 29521626

[10] Nojima T Reynolds AE Kitamura D Kelsoe G Kuraoka M. Tracing self-reactive b cells in normal mice. J Immunol (2020) 205:90–101. doi: 10.4049/jimmunol.1901015 32414809 PMC7311292

[11] Merrell KT Benschop RJ Gauld SB Aviszus K Decote-Ricardo D Wysocki LJ. Identification of anergic b cells within a wild-type repertoire. Immunity (2006) 25:953–62. doi: 10.1016/j.immuni.2006.10.017 17174121

[12] Duty JA Szodoray P Zheng NY Koelsch KA Zhang Q Swiatkowski M. Functional anergy in a subpopulation of naive b cells from healthy humans that express autoreactive immunoglobulin receptors. J Exp Med (2009) 206:139–51. doi: 10.1084/jem.20080611 PMC2626668 19103878

[13] Quach TD Manjarrez-Orduno N Adlowitz DG Silver L Yang H Wei C. Anergic responses characterize a large fraction of human autoreactive naive b cells expressing low levels of surface IgM. J Immunol (2011) 186:4640–8. doi: 10.4049/jimmunol.1001946 PMC3095097 21398610

[14] Burnett DL Reed JH Christ D Goodnow CC. Clonal redemption and clonal anergy as mechanisms to balance b cell tolerance and immunity. Immunol Rev (2019) 292:61–75. doi: 10.1111/imr.12808 31556969

[15] Noviski M Zikherman J. Control of autoreactive b cells by IgM and IgD b cell receptors: maintaining a fine balance. Curr Opin Immunol (2018) 55:67–74. doi: 10.1016/j.coi.2018.09.015 30292928 PMC6291015

[16] Zikherman J Parameswaran R Weiss A. Endogenous antigen tunes the responsiveness of naive b cells but not T cells. Nature (2012) 489:160–4. doi: 10.1038/nature11311 PMC3438375 22902503

[17] Ubelhart R Hug E Bach MP Wossning T Duhren-von Minden M Horn AH. Responsiveness of b cells is regulated by the hinge region of IgD. Nat Immunol (2015) 16:534–43. doi: 10.1038/ni.3141 25848865

[18] Sabouri Z Perotti S Spierings E Humburg P Yabas M Bergmann H. IgD attenuates the IgM-induced anergy response in transitional and mature b cells. Nat Commun (2016) 7:13381. doi: 10.1038/ncomms13381 27830696 PMC5109548

[19] Amendt T Ayoubi OE Linder AT Allies G Young M Setz CS. Primary immune responses and affinity maturation are controlled by IgD. Front Immunol (2021) 12:709240. doi: 10.3389/fimmu.2021.709240 34434193 PMC8381280

[20] Sabouri Z Schofield P Horikawa K Spierings E Kipling D Randall KL. Redemption of autoantibodies on anergic b cells by variable-region glycosylation and mutation away from self-reactivity. Proc Natl Acad Sci U.S.A. (2014) 111:E2567–75. doi: 10.1073/pnas.1406974111 PMC4078846 24821781

[21] Reed JH Jackson J Christ D Goodnow CC. Clonal redemption of autoantibodies by somatic hypermutation away from self-reactivity during human immunization. J Exp Med (2016) 213:1255–65. doi: 10.1084/jem.20151978 PMC4925023 27298445

[22] Di Niro R Mesin L Zheng NY Stamnaes J Morrissey M Lee JH. High abundance of plasma cells secreting transglutaminase 2-specific IgA autoantibodies with limited somatic hypermutation in celiac disease intestinal lesions. Nat Med (2012) 18:441–5. doi: 10.1038/nm.2656 PMC4533878 22366952

[23] IJspeert H van Schouwenburg PA van Zessen D Pico-Knijnenburg I Stubbs AP van der Burg M. Antigen receptor galaxy: A user-friendly, web-based tool for analysis and visualization of T and b cell receptor repertoire data. J Immunol (2017) 198:4156–65. doi: 10.4049/jimmunol.1601921 PMC5421304 28416602

[24] Gupta NT Vander Heiden JA Uduman M Gadala-Maria D Yaari G Kleinstein SH. Change-O: A toolkit for analyzing large-scale b cell immunoglobulin repertoire sequencing data. Bioinformatics (2015) 31:3356–8. doi: 10.1093/bioinformatics/btv359 PMC4793929 26069265

[25] Stern JN Yaari G Vander Heiden JA Church G Donahue WF Hintzen RQ. B cells populating the multiple sclerosis brain mature in the draining cervical lymph nodes. Sci Transl Med (2014) 6:248ra107. doi: 10.1126/scitranslmed.3008879 PMC4388137 25100741

[26] Boughter CT Borowska MT Guthmiller JJ Bendelac A Wilson PC Roux B. Biochemical patterns of antibody polyreactivity revealed through a bioinformatics-based analysis of CDR loops. Elife (2020) 9. doi: 10.7554/eLife.61393 PMC7755423 33169668

[27] Morbach H Eichhorn EM Liese JG Girschick HJ. Reference values for b cell subpopulations from infancy to adulthood. Clin Exp Immunol (2010) 162:271–9. doi: 10.1111/j.1365-2249.2010.04206.x PMC2996594 20854328

[28] Koelsch K Zheng NY Zhang Q Duty A Helms C Mathias MD. Mature b cells class switched to IgD are autoreactive in healthy individuals. J Clin Invest (2007) 117:1558–65. doi: 10.1172/JCI27628 PMC1866247 17510706

[29] Seifert M Steimle-Grauer SA Goossens T Hansmann ML Brauninger A Kuppers R. A model for the development of human IgD-only b cells: Genotypic analyses suggest their generation in superantigen driven immune responses. Mol Immunol (2009) 46:630–9. doi: 10.1016/j.molimm.2008.07.032 18952293

[30] Zheng NY Wilson K Wang X Boston A Kolar G Jackson SM. Human immunoglobulin selection associated with class switch and possible tolerogenic origins for c delta class-switched b cells. J Clin Invest (2004) 113:1188–201. doi: 10.1172/JCI20255 PMC385404 15085198

[31] Setz CS Khadour A Renna V Iype J Gentner E He X. Pten controls b-cell responsiveness and germinal center reaction by regulating the expression of IgD BCR. EMBO J (2019) 38(11). doi: 10.15252/embj.2018100249 PMC6545559 31015337

[32] Ubelhart R Jumaa H. Autoreactivity and the positive selection of b cells. Eur J Immunol (2015) 45:2971–7. doi: 10.1002/eji.201444622 26350303

[33] Maity PC Blount A Jumaa H Ronneberger O Lillemeier BF Reth M. B cell antigen receptors of the IgM and IgD classes are clustered in different protein islands that are altered during b cell activation. Sci Signal (2015) 8:ra93. doi: 10.1126/scisignal.2005887 26373673

[34] Enders A Short A Miosge LA Bergmann H Sontani Y Bertram EM. Zinc-finger protein ZFP318 is essential for expression of IgD, the alternatively spliced igh product made by mature b lymphocytes. Proc Natl Acad Sci U.S.A. (2014) 111:4513–8. doi: 10.1073/pnas.1402739111 PMC3970522 24616512

[35] Pioli PD Debnath I Weis JJ Weis JH. Zfp318 regulates IgD expression by abrogating transcription termination within the Ighm/Ighd locus. J Immunol (2014) 193:2546–53. doi: 10.4049/jimmunol.1401275 PMC4135021 25057009

[36] Kirchenbaum GA St Clair JB Detanico T Aviszus K Wysocki LJ. Functionally responsive self-reactive b cells of low affinity express reduced levels of surface IgM. Eur J Immunol (2014) 44:970–82. doi: 10.1002/eji.201344276 PMC3984621 24375379

[37] Tan C Mueller JL Noviski M Huizar J Lau D Dubinin A. Nur77 links chronic antigen stimulation to b cell tolerance by restricting the survival of self-reactive b cells in the periphery. J Immunol (2019) 202:2907–23. doi: 10.4049/jimmunol.1801565 PMC6504591 30962292

[38] Tull TJ Pitcher MJ Guesdon W Siu JHY Lebrero-Fernandez C Zhao Y. Human marginal zone b cell development from early T2 progenitors. J Exp Med (2021) 218(4). doi: 10.1084/jem.20202001 PMC7868795 33538776

[39] Vossenkamper A Blair PA Safinia N Fraser LD Das L Sanders TJ. A role for gut-associated lymphoid tissue in shaping the human b cell repertoire. J Exp Med (2013) 210:1665–74. doi: 10.1084/jem.20122465 PMC3754866 23940259

[40] Potter KN Hobby P Klijn S Stevenson FK Sutton BJ. Evidence for involvement of a hydrophobic patch in framework region 1 of human V4-34-encoded igs in recognition of the red blood cell I antigen. J Immunol (2002) 169:3777–82. doi: 10.4049/jimmunol.169.7.3777 12244172

[41] Zhang Z Jara CJ Singh M Xu H Goodnow CC Jackson KJ. Human transitional and IgM(low) mature naive b cells preserve permissive b-cell receptors. Immunol Cell Biol (2021) 99:865–78. doi: 10.1111/imcb.12478 PMC8453828 33988890

