# Supplementary material for: IgD shapes the pre-immune naïve B cell compartment in humans
Source: Front Immunol. 2023 Jan 26;14:1096019. doi: 10.3389/fimmu.2023.1096019 (PMC9908586; doi:10.3389/fimmu.2023.1096019)
Supplement: Supplementary file 1 [file DataSheet_1.pdf]

Supplementary Material

1 Supplementary Methods

The following antibodies were used for flow cytometric analysis and sorting: FITC anti-human IgD (polyclonal, Caltag), PE or PE-Cy7 anti-human IgD (clone IA6-2, Biolegend), Brilliant violet 421 anti-human IgM (clone MHM-88, Biolegend), PE anti-human kappa (polyclonal, DAKO), FITC anti-human lambda polyclonal, DAKO), PE-Cy7 anti-human CD10 (clone HI10a, Biolegend), APC-Cy7 anti-human CD19 (clone HIB19, Biolegend), PE-Cy7 anti-human CD20 (clone 2H7), APC anti-human CD21 (Bu32), FITC anti-human CD22 (S-HCL-1, Biolegend, FITC anti-human CD24 (clone ML5, Biolegend), PerCP-Cy5.5 anti-human CD27 (M-T271, Biolegend), PerCP-Cy5.5 anti-human CD34 (clone 561, Biolegend), FITC or Brilliant Violet 421 anti-human CD38 (HIT2, Biolegend), PE anti-human CD40 (clone 5C3, Biolegend), PE-Cy7 anti-human CD69 (clone FN50, Biolegend), APC anti-human CD86 (clone IT2.2, Biolegend), PE anti-human HLA-DR (clone 1.243, Biolegend), FITC anti-human BAFF-R (clone 11C1, Biolegend), PE anti-human TACI (clone 1A1, Biolegend), PE anti-human CXCR4 (clone QA18A64, Biolegend), APC anti-human integrin β (clone FIB504, Biolegend).

2 Supplementary Tables

Table S1 – Immunological parameters in individuals carrying a heterozygous IGHD variant

	**K1-I.1**	**K1-II.2**	**K1-II.3**	**K1-III.1**	**K1-III.3**	**K2-I.1**	**K2-II.1**	**K3-II.1**
Age (years) / sex	65 / male	42 / female	38 / male	8 / female	5 / female	63 / male	32 / male	9 / male
Disease	healthy	healthy	healthy	healthy	CVID	healthy	Hodgkin`s lymphoma	healthy
IGHD variant	p.P6L	p.P6L	p.P6L	p.P6L	p.P6L	p.C15VfsX21	p.C15VfsX21	p.L328PfsX389
WBC (/µl)	5,850	7,980	8,570	6,790	13,300	n.a.	3,000 (-)	7.650
Lymphocytes (/µl)	1,790	2,100	2,190	2,320	3,830	n.a.	1,220 (-)	2.600
$CD3^+$ (%)	59.3	78.3	62.2	73.9	64.4	n.a.	62.6	76.1
$CD3^+$ (/µl)	1,062	1650	1,359	1716	2166	n.a.	759 (-)	1,985
$CD3^+CD4^+$ (%)	43.6	50.5	45.2	33.6	33.6	n.a.	28.7	49.5
$CD3^+CD4^+$ (/µl)	780	1064	987	779	1,131	n.a.	347 (-)	1,292
$CD3^+CD8^+$ (%)	10.2	22.6	14.6	30.4	25.4	n.a.	27.9	21.4
$CD3^+CD8^+$ (/µl)	183	475	319	705	856	n.a.	338 (-)	558
$CD3^-CD56^+$ (%)	33.4	12.1	27.2 (+)	14.7	9.2	n.a.	27.6	2.4
$CD3^-CD56^+$ (/µl)	597	255	604 (+)	342	614	n.a.	335	63 (-)
$CD3^-CD19^+$ (%)	4.6	5.5	14.2	5.7	18.3	n.a.	8.5	18.4
$CD3^-CD19^+$ (/µl)	82	116	92	133	614	n.a.	103	487
$CD27^+/CD19^+$ (%)	31.4	28.6	38.5	31.0 (+)	0.6 (-)	n.a.	2.5 (-)	17.3
$CD27^+/CD19^+$ (µl)	26	33	35	41	5	n.a	3	84
$CD27^-IgD^+/CD19^+$ (%)	31.1	44.7	38.4	43.4	60.9	n.a.	51.8	47.1

$CD27^-IgD^+/CD19^+$ (µl)	25	52	35	46	373	n.a.	53	229
$CD27^-IgD^-/CD19^+$ (%)	37.5	26.7	23.1	25.6	38.5	n.a.	45.7	35.6
$CD27^-IgD^-/CD19^+$ (µl)	31	31	22	34	236	n.a.	47	172

n.a., not assessed; (+) above reference value; (-) below reference value

	**K1-I.1**	**K1-II.2**	**K1-II.3**	**K1-III.1**	**K1-III.3**	**K2-I.1**	**K2-II.1**	**K3-II.1**
IgD (mg/dl)	<0.6	<0.6	<0.7	1.0	<0.7	<0.6	<0.6	n.a.
IgM (mg/dl)	94	54	43	70	38 (-)	112	181	65
IgA (mg/dl)	159	80	106	74	6 (-)	440	166	120
IgG (mg/dl)	1054	993	1162	1040	280 (-)	1610	1054	841
IgG1 (mg/dl)	468	423	586	748	110	n.a.	547	n.a.
IgG2 (mg/dl)	428	477	499	247	64	n.a.	239	n.a.
IgG3 (mg/dl)	83	66	75	51	107	n.a.	83	n.a.
IgG4 (mg/dl)	16	36	28	19	<0.3	n.a.	4.4 (-)	n.a.
Tetanus-Toxoid IgG Antibody (U/ml)	2.78	0.94	4.54	0.32	0.04 (-)	>5.0	0.16	n.a.
Diphteria-Toxoid IgG Antibody (U/ml)	0.16	0.34	0.78	0.47	0.08 (-)	0.59	0.05 (-)	n.a.
Mumps Antibody IgG Antibody (titre)	1:4,500	1:1,000	1:2,500	1:740	<1:230 (-)	1:920	1:1,100	n.a.
Measles Antibody IgG Antibody (mU/ml)	10.000	8,200	3,400	540	150 (borderline)	9.800	230 (borderline)	n.a.
Rubella Antibody IgG Antibody (U/ml)	150	150	54	40	15	23	7	n.a.

Varicella Antibody IgG Antibody (mU/ml)	1.400	760	410	220	<50 (-)	250	670	n.a.

n.a., not assessed; (+) above reference value; (-) below reference value

Table S2 – Sorted cell numbers used for IgH sequencing and obtained sequences

**Individuum**	**Sorted Population**	**Number of sorted cells**	**Overall reads**	**Number of unique CDR3**	**IgM (%)**	**IgD (%)**	**IgA/IgG/IgE (%)**
K1-III.1	IgD+	$1.9*10^4$	288802	9407	72.22	27.76	0.00
	IgD-	$1.6*10^4$	285303	14743	69.12	30.21	0.66
K1-II.3	IgD+	$1.1*10^5$	102283	24043	76.59	23.39	0.02
	IgD-	$6.8*10^4$	101893	15521	80.99	18.91	0.10
K2-I.1	IgD+	$6.3*10^4$	98168	14346	72.61	27.38	0.02
	IgD-	$5.3*10^4$	104427	10973	96.83	3.17	0.01
K3-II.1	IgD+	$3.5*10^5$	102442	48866	81.53	18.45	0.01
	IgD-	$2.7*10^5$	92160	36435	90.83	9.11	0.05

IgD^+ population: $CD19^+CD27^-CD10^+IgM^+$ IgD^+; IgD^- population: $CD19^+CD27^-CD10^+IgM^+$ IgD^-

3 Supplementary Figures

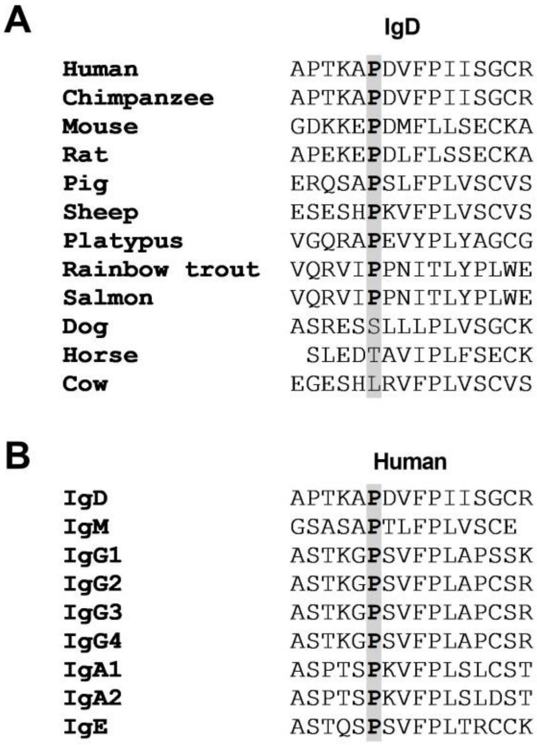

Figure S1 – Evolutionary conservation of IgD

Alignment (A) of IgD amino acid sequences between different species and (B) of different Ig isotypes in humans. The amino acid position 6 is highlighted in grey.

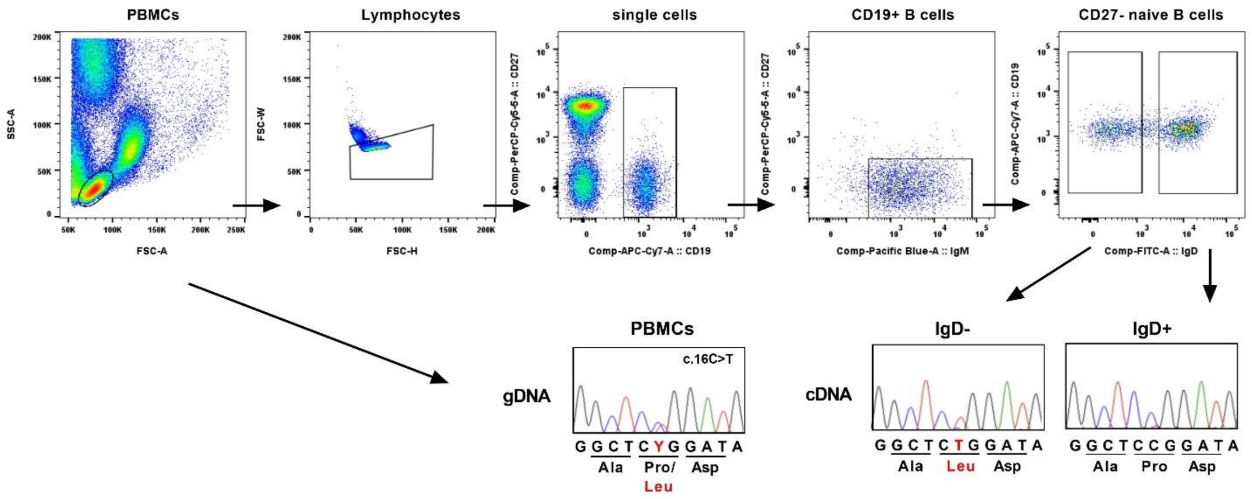

Figure S2 – Usage of different *IGHD* alleles in IgD+ and IgD- naïve B cell populations

Dot blots showing the gating strategy for sorting IgD^{+} and IgD^{-} CD19^{+}CD27^{-}IgM^{+} naïve B cells from individual K1-III.3. Chromatograms showing sequencing results of *IGHD* exon 1 amplified from cDNA of sorted B cell populations or genomic DNA (gDNA) from unsorted PBMCs.

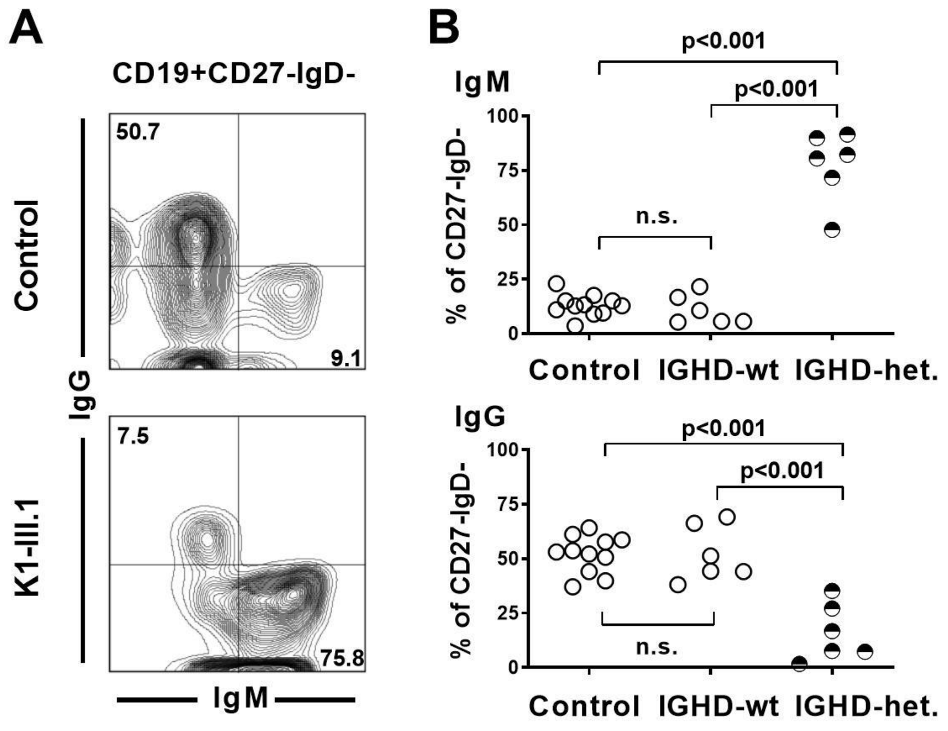

Figure S3 – IgD-CD27- B cells in heterozygous *IGHD* variant carriers are naïve B cells

(A) Representative dot plots showing IgG and IgM expression in $CD19^{+}CD27^{-}IgD^{-}$ B cells from an individual carrying an *IGHD* variant and an unrelated control individual. (B) Frequencies of IgM- or IgG-positive cells within $CD19^{+}CD27^{-}IgD^{-}$ B cells from individuals carrying a heterozygous *IGHD* variant (IGHD-het.), their family members without *IGHD* variant (*IGHD*-wt) and unrelated control individuals.

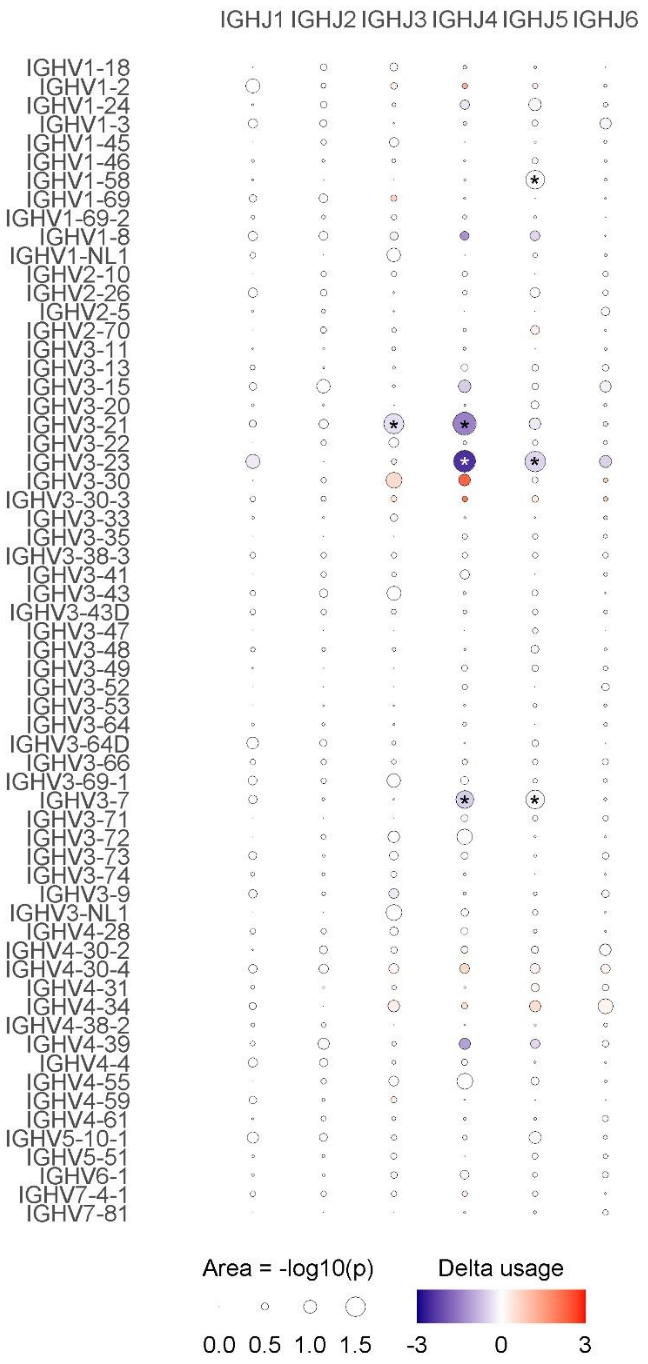

Figure S4 – Distribution of V_H-J_H rearrangements in IgD+ and IgD- naïve B cell populations

Differential usage of distinct V_H-J_H rearrangements compared between IgD^+ and IgD^- naïve B cell populations is shown from four individual donors. Mean difference is indicated by color and p values determined by paired t-test are indicated by area of the circles (* $p<0.05$).

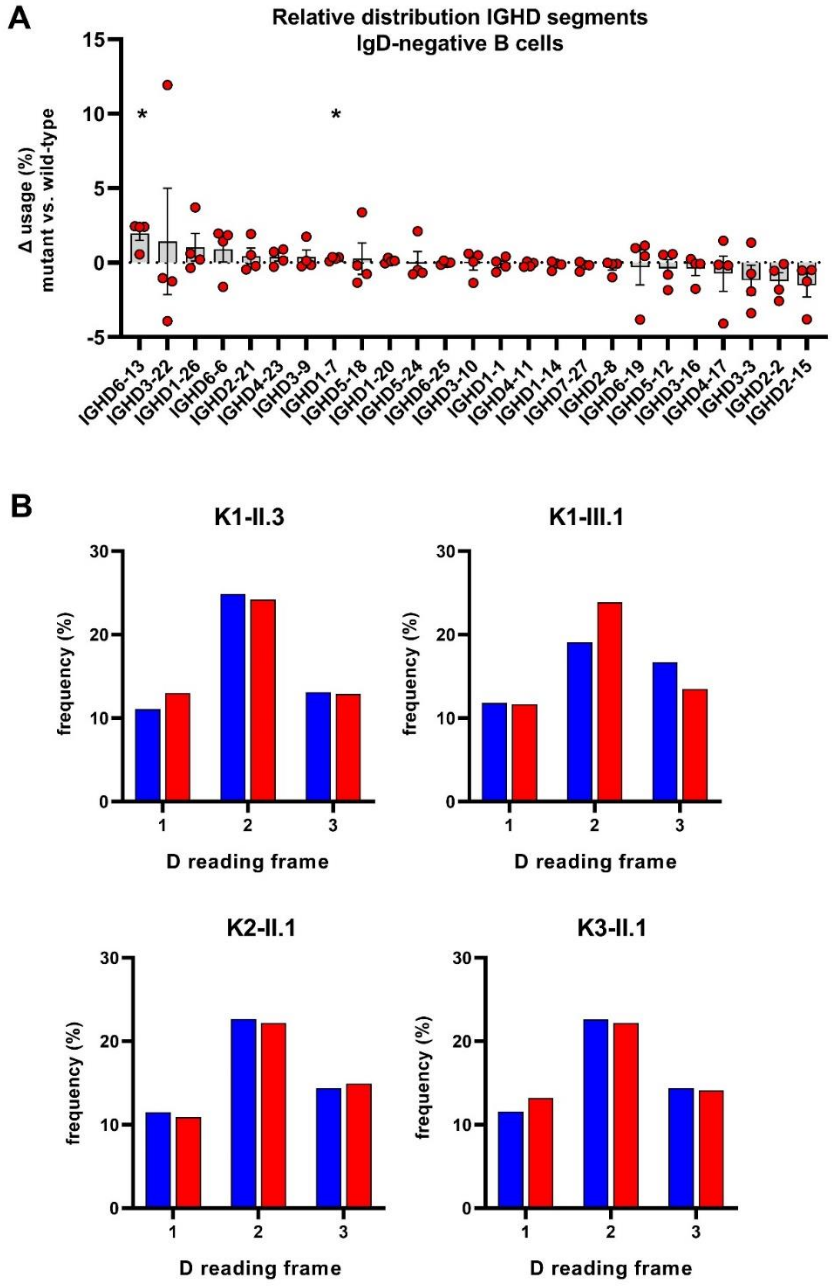

Figure S5 – D_H segments and D_H reading frame

(A) Distribution of different DH segments used in CD19$^+$CD27$^-$CD21$^+$CD10$^-$IgM$^+$IgD$^-$ mature naïve B cells is shown as relative difference compared to the IgD+ counterpart. Symbols represent the Δ frequency IgD$^-$/mutant versus IgD$^+$/wild-type from individual samples and bars the mean ± SEM. DH segments are ordered on the x-axis according to their mean relative difference. (B) The frequency of the D reading frame used in IgD$^+$ (blue) and IgD$^-$ (red) B cell subsets is shown for each individual.

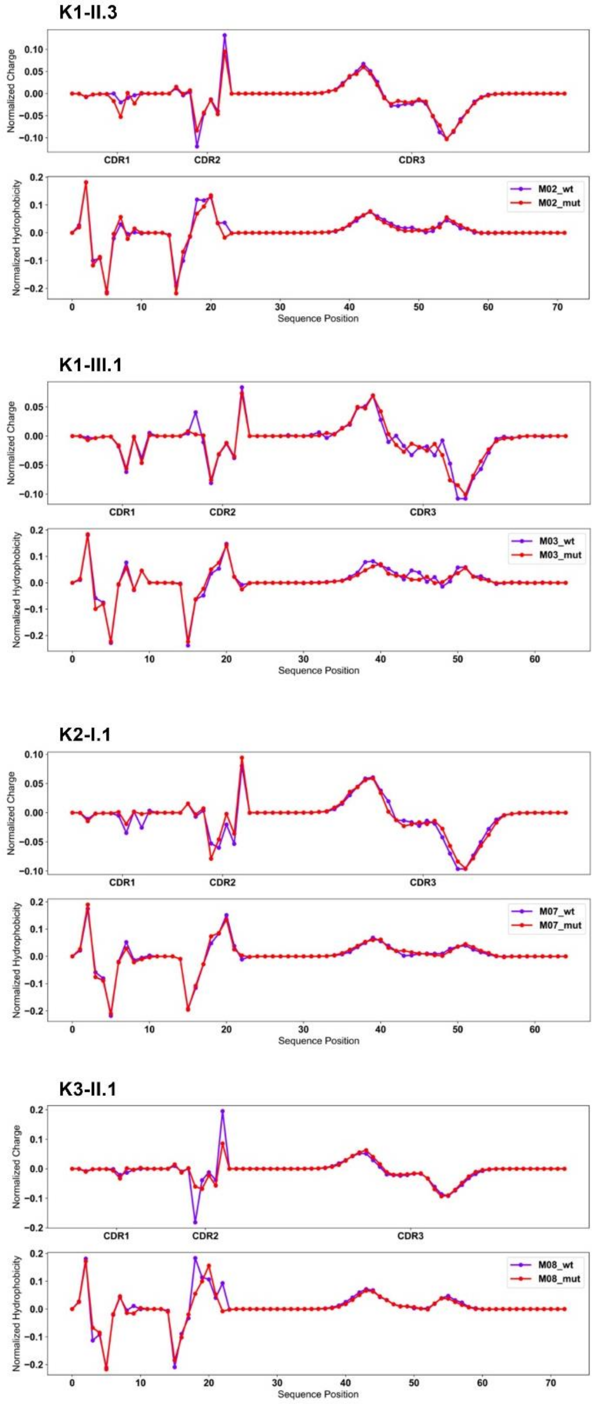

<u>Figure S6 – Position sensitive IGH CDR properties of IgD+ and IgD- naïve B cell populations</u>

Position sensitive analysis of normalized charge and hydrophobicity in CDRs of IGH sequences from IgD^+ (blue line) and IgD^- (red line) naïve B cells. Position sensitive properties are shown as analyzed by AIMS for each patient separately.

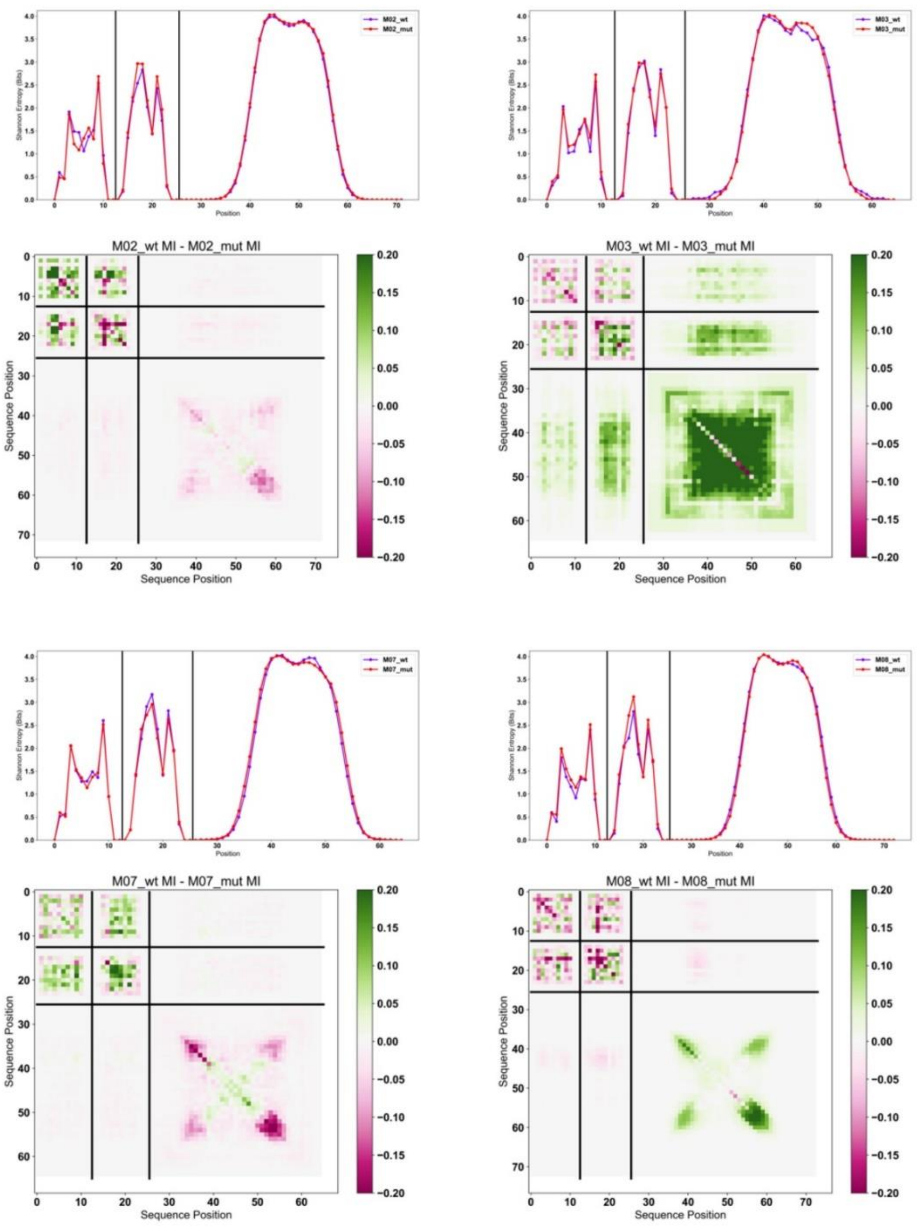

Figure S7 –Shannon entropy and mutual information difference of the IgH sequences between IgD+ and IgD- naïve B cells

Position sensitive analysis of Shannon entropy in CDRs of IGH sequences from IgD$^+$ (blue line) and IgD$^-$ (red line) naïve B cells (upper panels). Differences in mutual information of IgH sequences were compared between IgD$^+$ and IgD$^-$ naïve B cells for each individual donor (lower panels). Individual rows within the squares represent the given condition and columns the location of the calculated mutual information. Green is indicating a higher mutual information in the IgD$^+$ population and purple in the IgD$^-$ population.
